# SPARSE: a sparse hypergraph neural network for learning multiple types of latent combinations to accurately predict drug–drug interactions

**DOI:** 10.1093/bioinformatics/btac250

**Published:** 2022-06-27

**Authors:** Duc Anh Nguyen, Canh Hao Nguyen, Peter Petschner, Hiroshi Mamitsuka

**Affiliations:** Bioinformatics Center, Institute for Chemical Research, Kyoto University, Uji, Japan; Bioinformatics Center, Institute for Chemical Research, Kyoto University, Uji, Japan; Bioinformatics Center, Institute for Chemical Research, Kyoto University, Uji, Japan; Department of Pharmacodynamics, Semmelweis University, Budapest, Hungary; Bioinformatics Center, Institute for Chemical Research, Kyoto University, Uji, Japan; Department of Computer Science, Aalto University, Espoo, Finland

## Abstract

**Motivation:**

Predicting side effects of drug–drug interactions (DDIs) is an important task in pharmacology. The state-of-the-art methods for DDI prediction use hypergraph neural networks to learn latent representations of drugs and side effects to express high-order relationships among two interacting drugs and a side effect. The idea of these methods is that each side effect is caused by a unique combination of latent features of the corresponding interacting drugs. However, in reality, a side effect might have multiple, different mechanisms that cannot be represented by a single combination of latent features of drugs. Moreover, DDI data are sparse, suggesting that using a sparsity regularization would help to learn better latent representations to improve prediction performances.

**Results:**

We propose SPARSE, which encodes the DDI hypergraph and drug features to latent spaces to learn multiple types of combinations of latent features of drugs and side effects, controlling the model sparsity by a sparse prior. Our extensive experiments using both synthetic and three real-world DDI datasets showed the clear predictive performance advantage of SPARSE over cutting-edge competing methods. Also, latent feature analysis over unknown top predictions by SPARSE demonstrated the interpretability advantage contributed by the model sparsity.

**Availability and implementation:**

Code and data can be accessed at https://github.com/anhnda/SPARSE.

**Supplementary information:**

Supplementary data are available at *Bioinformatics* online.

## 1 Introduction

A drug-drug interaction (DDI) is a reaction between two drugs, whereby the effects of one drug are modified by the concomitant use of the second drug. A DDI might cause side effects, which are unwanted effects and are responsible for significant patient morbidity and mortality (Magro *et al.*, 2012). Hence, predicting side effects of a DDI, i.e. DDI prediction, is a very important task to guarantee drug safety.

Using machine learning has emerged as a prominent approach for DDI prediction, making the prediction fast and highly accurate (Xu *et al.*, 2019; Zitnik *et al.*, 2018). The traditional machine learning methods such as support-vector machines (Kastrin *et al.*, 2018), logistic regression (Mei and Zhang, 2021) or feedforward neural networks (Wang *et al.*, 2019) use predefined drug features to predict side effects as labels. However, DDI data have more information. Particularly, DDIs can be represented by a graph, called a DDI graph, where nodes are drugs and edges are interacting drugs. The DDI graph can be learned with graph neural networks (Zitnik *et al.*, 2018). Nonetheless, DDI graphs are only limited to pairwise relationships of drug pairs while there still exist many side effects, which can be represented by other relationships, such as co-occurrence. Then, a state-of-the-art generalization of a DDI graph can be a DDI hypergraph, which can capture higher-order relationships, where drugs and side effects are both nodes, and each hyperedge is a triple of a side effect with two interacting drugs.

On the DDI hypergraph, hypergraph neural networks can be applied to learn the representations of drugs and side effects altogether. In DDIs, two drugs with totally different properties can still interact with each other, hence the traditional hypergraph neural networks using similarity assumption on node representations are not suitable (Feng *et al.*, 2019). Instead, CentSmoothie, a current cutting-edge hypergraph neural network for DDIs (Nguyen *et al.*, 2021), assumes that each side effect is caused by a unique combination of latent features of the corresponding interacting drugs. However, in real life, each side effect might have many different mechanisms (Suleyman *et al.*, 2010) that cannot be reflected in a single combination of drug latent features. Hence, it is necessary to learn different types of combinations of drug latent features for each side effect. This is the first problem **(P1)**, which we address in this article.

To solve P1, we borrow one idea of stochastic block models (SBMs) on hypergraphs such that each node (e.g. drug or side effect) has one or several latent features (Anandkumar *et al.*, 2013; Pal and Zhu, 2021) and there exist interactions (associations) of latent features. This method can learn different types of combinations of drug latent features for each side effect, at once. In addition, to improve the quality of learned latent features, input node features also can be used (Zhang *et al.*, 2019). However, transformations from input node features and node relationships in the hypergraphs to latent features might be complex and, especially, non-linear. This is the second problem **(P2)**, which has not been addressed in existing SBMs and we address in this article.

Moreover, DDI data are sparse (e.g. in the largest DDI dataset, 97.6% of all triples of drug–drug-side effects are not a DDI), suggesting that the model for learning DDIs also should be sparse. However, recent work on DDIs has not used this sparsity of the data (Nguyen *et al.*, 2021; Zitnik *et al.*, 2018), which might potentially impair model performance. This is the third problem (**P3**), which we address in this article.

**Fig. 1.**
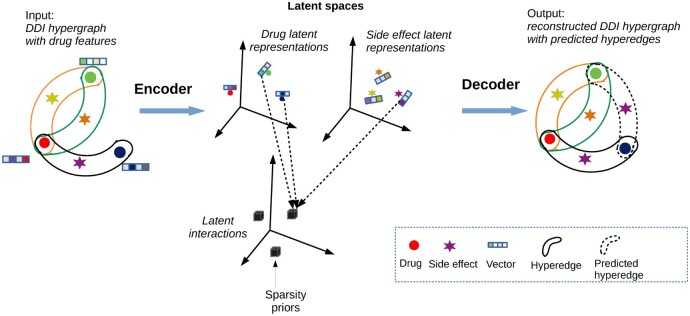
A schematic illustration of the procedure in the proposed model, SPARSE

We propose SPARSE, a new model for DDI prediction, to solve the above three problems. For P1, we assume that there exist drug and side effect latent features with latent interactions so that each side effect latent feature interacts with several pairs of drug latent features. For P2, we encode drug features and the DDI hypergraph altogether in the latent representations using a suitable hypergraph neural network. For P3, we guide the model to preserve the sparsity of the data using a suitable sparsity control. Figure 1 schematically illustrates these ideas of our model. That is, the model consists of two parts: (i) an encoder and (ii) a decoder. The encoder encodes the input of the DDI hypergraph (e.g. three hyperedges in Fig. 1) with drug features into latent spaces of drug and side effect latent representations, and interactions of latent features. The decoder reconstructs from the latent spaces the DDI hypergraph with new DDI predictions (e.g. the dotted hyperedge in Fig. 1). Finally, a sparsity prior (horseshoe priors in our model) is used to control the sparsity of the latent interactions.

Our extensive experiments first validated the advantage of SPARSE in terms of prediction performance by using both synthetic and real-world datasets. Throughout all experiments on prediction performance, SPARSE achieved better prediction performances than competing methods, such as CentSmoothie and SBM. For example, in the experiment of using the largest real DDI dataset, called TWOSIDES, SPARSE achieved area under the ROC curve (AUC) of 0.9524 and (area under the precision-recall curve (AUPR) of 0.882, while CentSmoothie achieved AUC of 0.9348 and AUPR of 0.8749 and SBM achieved AUC of 0.9337 and AUPR of 0.8583. Similarly when using JADERDDI, another DDI dataset, SPARSE achieved AUC of 0.9698 and AUPR of 0.7348, while CentSmoothie was AUC of 0.9684 and AUPR of 0.6044 and SBM was AUC of 0.9428 and AUPR of 0.5963.

We then examined the top prediction obtained by SPARSE, which is trained by using the whole TWOSIDES. That is, we checked the number of overlaps between the top 400 predictions by one method and DDIs in drugs.com (Drugs.com, 2021; Thelwall *et al.*, 2017), which is a commonly used online web checker for DDI. We found 98 DDIs in drugs.com out of the top 400 predictions, while by using the same procedure, CentSmoothie found only 71 DDIs out of the top 400 predictions, implying that SPARSE can find new DDIs more than competing methods.

Finally, we validated the prediction results by characterizing the top predictions obtained by SPARSE. In more detail, we checked the biological properties, such as target proteins, of the top 10 triples of drug–drug-side effect, predicted by SPARSE, by using latent features connected to these top 10 predictions. We then found that top predictions can be associated with some biological mechanisms and particularly with responsible proteins/pathways. These results indicate that our model, SPARSE, can provide high predictive performances as well as latent biological knowledge beneficial to understand the background behind predicted DDIs.

## 2 Related work

Machine-learning models for DDI prediction can be divided into non-graph-based and graph-based ones. For non-graph-based models, the inputs are the predefined feature vectors of pairs of drugs, the outputs are the corresponding side effects, and the models are multi-label classifiers, e.g. support-vector machines (Kastrin *et al.*, 2018) or a multilayer feedforward neural network (Wang *et al.*, 2019). Instead of only using predefined drug feature vectors, graph-based methods for DDI use graph neural networks to learn new latent representations of drugs from molecular graphs or DDI graphs. In molecular graphs, each drug is considered as a graph that nodes are atoms and edges are connections of atoms (Harada *et al.*, 2020; Xu *et al.*, 2019). In DDI graphs, DDIs are considered as pairwise relationships and formulated in the form of a graph where nodes are drugs and edges are drug interactions with side effects as labels (Zitnik *et al.*, 2018). The latter one has shown to be more effective for DDI prediction since it can use both pharmacological information and biological information rather than only molecular graphs (Zitnik *et al.*, 2018).

However, one drawback of using graph neural networks on DDI graphs is that it does not use multiple relationships (labels) at the same time. Side effects themselves have relationships with each other, e.g. co-occurrences. Existing work often fixes them as one-hot vectors to indicate the presence of the side effects. This representation considers side effects independently, potentially making the models under-utilize the side effect relationships.

Hypergraph neural networks on DDI overcome the above drawback by learning representations of drug and side effect nodes altogether in latent spaces (Nguyen *et al.*, 2021). DDI is considered as high-order relationships of drug–drug-side effects in the form of a hypergraph where nodes are both drugs and side effects, and each hyperedge is a triple of two interacting drugs and a side effect caused by the drugs. There are two types of hypergraph neural networks models on the DDI: similarity based and non-similarity based. The similarity-based models, e.g. traditional spectral-based hypergraph neural networks, assume that interacting drugs should have similar representations (Fan *et al.*, 2021; Feng *et al.*, 2019). However, in DDI, two interacting drugs are not necessarily similar. For non-similarity models, the current state-of-the-art method is CentSmoothie (Nguyen *et al.*, 2021) that assumes that the representation of a side effect can be represented by a combination of latent features of two drugs causing the side effect. However, CentSmoothie cannot deal with multiple combinations of latent features at the same time.

In order to deal with multiple combinations of latent features, one possible approach is to use the idea of SBMs, which can be applied to hypergraphs, with each node belonging to several latent features (groups) and associations of latent features (groups) (Anandkumar *et al.*, 2013). However this has not been applied to DDI hypergraphs, and more importantly, SBM is based on linear assumption, while DDI can be generated through more complex relations to be represented by non-linearity.

Many studies have shown the benefits of sparsity regularization, which is a commonly used method to achieve sparsity of models, especially on noisy and sparse data (Carvalho *et al.*, 2009; Tibshirani, 1996). In a Bayesian viewpoint, sparsity regularization can be understood as a result of using sparse prior distributions. A state-of-the-art method for sparsity regularization is to use horseshoe priors (Carvalho *et al.*, 2009; Piironen and Vehtari, 2017). It shows an advantage in comparison with traditional Laplace prior (Lasso regularization) (Tibshirani, 1996) in that the horseshoe prior allows to shrink in both directions: no shrinkage for important features and complete shrinkage for non-important (noise) features. A comparable shrinkage prior with the horseshoe prior is the spike-and-slab prior (Hoeting *et al.*, 1999). However, the spike-and-slab prior is a discrete prior that requires the Markov chain Monte Carlo sampling for optimization, which is not effective for large-scale datasets like DDI.

## 3 Materials and methods

### 3.1 Background

We recall definitions for horseshoe priors and *n*-mode tensor product for 3D tensors, which will be used later.

#### 3.1.1 Horseshoe priors

We summarize the horseshoe prior (Carvalho *et al.*, 2009), a state-of-the-art prior for sparsity control, for a non-negative 3D tensor: $B=\{B_{i,j,k}\}\in R_{0+}^{K_{1}\times K_{2}\times K_{3}}$. The idea of the horseshoe prior is that each $B_{i,j,k}$ follows a normal distribution with the same zero mean and a different variance. Each variance has two parts: one is a global parameter sharing among all variances to decide the sparsity of **B** and one is a local parameter to decide the magnitude of each variance by using a heavy tail distribution with the half-Cauchy distribution. In more detail:

(1) $$B_{i,j,k}∼N(0,\tau^{2}\Lambda_{i,j,k}^{2}),$$

 

(2) $$\Lambda_{i,j,k}∼C^{+}(0,1),$$

where *τ* is a global parameter for sparsity, and $C^{+}(0,1)$ is a half-Cauchy distribution defined by: $p(\Lambda_{i,j,k})=\frac{2}{\pi }\frac{1}{1+\Lambda_{i,j,k}^{2}}$ for $\Lambda_{i,j,k}\geq 0$.

Both the horseshoe prior and Laplace prior (for Lasso regularization) are shrinkage priors such that by using priors, values of features tend to be shrunk (Piironen and Vehtari, 2017). Let ${\hat{B}}_{i,j,k}$ be the optimal values without priors, then the optimal values having priors has the form: ${\bar{B}}_{i,j,k}=(1-\kappa_{i,j,k}){\hat{B}}_{i,j,k}$, where $0\leq \kappa_{i,j,k}\leq 1$ is a shrinkage factor depending on the priors. With Laplace prior (Lasso regularization), the density of $\kappa_{i,j,k}$ tends to be a constant near 1 and disappears near 0, meaning that it always shrinks all features, containing important ones. In contrast, the density of $\kappa_{i,j,k}$ with the horseshoe prior has two peaks at 0 and 1, meaning that the horseshoe prior allows two kinds of shrinkage: no shrinkage to maintain important features and complete shrinkage to remove unimportant features.

#### 3.1.2 *N*-mode tensor product

The *n*-mode tensor product can be understood as a generalization of the matrix dot product in high-dimension that the product is processed at the *n*th dimension. Considering in the 3D space with a tensor: $B\in R^{K_{1}\times K_{2}\times K_{3}}$ and a matrix $H\in R^{T\times K_{n}},n\in \{1,2,3\}$, the *n*-mode product of **B** and **H** is denoted by $B\times_{n}H$ and is defined for each of *n *=* *1, 2 and 3, as follows:

(3) $${\left(B\times_{1}H\right)}_{t,j,k}=\sum_{i=1}^{K_{1}}B_{i,j,k}H_{t,i}|t=1\ldots T,j=1\ldots K_{2},k=1\ldots K_{3},$$

 

(4) $${\left(B\times_{2}H\right)}_{i,t,k}=\sum_{j=1}^{K_{2}}B_{i,j,k}H_{t,j}|t=1\ldots T,i=1\ldots K_{1},k=1\ldots K_{3},$$

 

(5) $${\left(B\times_{3}H\right)}_{i,j,t}=\sum_{k=1}^{K_{3}}B_{i,j,k}H_{t,k}|t=1\ldots T,i=1\ldots K_{1},j=1\ldots K_{2}.$$

### 3.2 Problem formulation: DDI prediction

We formulate the DDI prediction problem as follows.

Input: Given a DDI hypergraph: $G=(V,E),V=V_{D}\cup V_{S},E\subset V_{D}\times V_{D}\times V_{S}$, where *V_D_* is a set of drug nodes, *V_S_* is a set of side effect nodes (given $u,v\in V_{D},\;t\in V_{S}$, two triples (*u*, *v*, *t*) and (*v*, *u*, *t*) are the same). The drug node features are $F_{D}\in R_{0+}^{|V_{D}|\times K_{0}}$ and the side effect node features are one-hot vectors: $F_{S}\in R_{0+}^{\left|V_{S}|\times |V_{S}\right|}.$

Output: For $e=(u,v,t)\in V_{D}\times V_{D}\times V_{S}$, calculate a prediction score for interaction *m*(*e*).

### 3.3 Proposed model

We propose SPARSE: a sparse model for learning multiple types of latent combinations of side effects and drugs to predict DDIs. Our model follows an auto-encoder framework with two parts: an encoder and a decoder. The encoder encodes the DDI hypergraph with drug node features to latent spaces with latent representations of drugs and side effects (**H**), and interactions of latent features (**B**). The decoder aims to reconstruct the DDI hypergraph with new predicted hyperedges from **H** and **B**. In the following parts, we first present our latent interaction assumption with sparsity for the interactions of drugs and side effects, and then we describe the encoder and decoder.

#### 3.3.1 Latent interaction assumption

To model DDIs, we suppose that there exist latent spaces with drug latent features and side effect latent features where DDIs occur. The latent interaction assumption is that two interacting drugs cause a side effect if there exist a pair of drug latent features of the two drugs that interact with a latent feature of the side effect.

In detail, the formulation for the latent interaction assumption can be described as follows. Let $L_{D}=\{1,\ldots ,K_{D}\}$ and $L_{S}=\{1,\ldots ,K_{S}\}$ be the sets of indices of latent features of drugs and side effect with *K_D_* and *K_S_* be the numbers of latent features. Let $B\in R_{0+}^{K_{D}\times K_{D}\times K_{S}}$ be a 3D tensor representing interactions of latent features of drugs and side effects. The set of interacting latent features is: $A=\{(i,j,k)\in L_{D}\times L_{D}\times L_{S}|B_{i,j,k}>0\}$.

Considering a triple of two drugs and one side effect $e=(u,v,t)\in V_{D}\times V_{D}\times V_{S}$. Let $h^{d}(u),h^{d}(v)\in R_{0+}^{K_{D}},\;h^{s}(t)\in R_{0+}^{K_{S}}$ be the vectors representing the presence of latent features of the two drugs and the side effect, respectively. Let $g_{u}=\{i\in L_{D}|h^{d}{\left(u\right)}_{i}>0\},\;g_{v}=\{i\in L_{D}|h^{d}{\left(v\right)}_{i}>0\}$ and $g_{t}=\{i\in L_{S}|h^{s}{\left(t\right)}_{i}>0\}$ be the sets of latent features of *u*, *v* and *t*, respectively.

Under the latent interaction assumption, *u* interacts with *v* to cause *t* if:

(6) $$g_{u}\times g_{v}\times g_{t}\cap A\neq \emptyset ,$$

or with tensor product formulation:

(7) $$B\times_{1}h^{d}(u)\times_{2}h^{d}(v)\times_{3}h^{s}(t)>0.$$

In practice, we can change the value 0 on the right side of Equation (7) to a positive threshold. Equation (6) will be used to generate synthetic data in the experimental section. Equation (7) will be used in the decoder of the model.

**Sparsity property**

We first define formulations for sparsity measures of the DDI data and the latent interactions using the percentages of non-interactions. Let *s_d_* be the sparsity of the hypergraph *G*:

(8) $$s_{d}=1-\frac{2|E|}{\left|V_{D}|(|V_{D}|-1)|V_{S}\right|}.$$

The sparsity of the latent interactions *s_l_* is defined as the percentage of the number of non-interacting triples of the latent features per the total number of all triples of the latent features.

(9) $$s_{l}=1-\frac{2|A|}{\left|L_{D}{|}^{2}|L_{S}\right|}.$$

DDI data are sparse as per statistics in Table 1. It is shown that 97.6% and 99.87% of all triples are non-interacting in TWOSIDES and JADERDDI, respectively.

**Table 1.** Statistics of three real datasets

Dataset	No. of Drugs	No. of Side effects	No. of Drug–drug pairs	No. of Drug–drug-side effects (DDIs)	Avg. no. of side effects/No. ofdrug–drug pairs	Sparsity (%)
TWOSIDES	557	964	49,677	3 606 046	72.58	97.6
CADDDI	587	969	21,918	373 976	17.06	99.77
JADERDDI	545	922	36,929	222 081	6.01	99.83

The motivation for us to use sparse models is that sparse models, according to statistical learning theory, are usually more reliable models if they could fit the training data well (Hastie, 2015). As our sparse models have sparse interactions among latent features, we will prove that they tend to generate sparse data and are suitable for DDI data. We show a relationship between sparsity of the models and sparsity of data generated by the models, which are the ones that best fit the models, as follows.

Property 1: Assume that the DDI data are generated from the true generation model according to formula (7). Assuming that each drug and side effect has exactly *n_u_* and *n_t_* non-zero latent features, respectively. Then, there exists a relationship between the sparsity of the model and the expected sparsity of the generated data as follows:

(10) $$E(s_{d})=1-(1-s_{l})\frac{n_{u}^{2}n_{t}}{K_{D}^{2}K_{S}}.$$

*Proof:*

For a pair of drug *u*, *v* to cause side effect *t*, then $B\times_{1}h^{d}(u)\times_{2}h^{d}(v)\times_{3}h^{s}(t)>0.$ This means that there is at least one non-zero entry of *B* corresponding to latent features of *u*, *v* and *t*. Since there are exactly $n_{u}^{2}n_{t}$ possible entries of B corresponding latent features of *u*, *v* and *t*, then the probability of a uniform sampling of entries of *B* to corresponding to these latent features is $p_{1}=\frac{n_{u}^{2}n_{t}}{K_{D}^{2}K_{S}}$. This is the probability of having an interaction among the features (that generates a side effect data point).

Since entries of *B* are assumed to be randomly sampled according to a uniform distribution, the number of interactions when **B** have $|B{|}_{0}=(1-s_{l})K_{D}^{2}K_{S}$ non-zero entries follows a binomial distribution $\mathrm{Binomial}(|B{|}_{0},p_{1})$.

With the assumption that the hypergraph is generated from this generative process, the expected number of non-zero data points (the number of hyperedges) becomes $|B{|}_{0}.p_{1}=(1-s_{l}).n_{u}^{2}n_{t}$. The expected sparsity of the hypergraph becomes $E(s_{d})=1-\frac{(1-s_{l})n_{u}^{2}n_{t}}{K_{D}^{2}K_{S}}=1-(1-s_{l})p_{1}$.

This result leads to $E(s_{d})>s_{l}p_{1}$. It shows a relationship between the sparsity of the model (*s_l_*) and the expected sparsity of the data generated by the model ($E(s_{d})$). It shows that the model can be sparse but cannot be as sparse as we want. It can be a hint on setting sparsity of the model in learning processes.

#### 3.3.2 Encoder

For the encoder, we use a hypergraph neural network with message passing (Yadati, 2020) to encode the input hypergraph and node features into latent spaces with node latent representations **H** and latent interactions **B** (for simplicity, **B** can be considered as a free parameter to learn).

(11) $$H=(H^{d},H^{s})=g_{w_{0}}(G,F)\in R_{0+}^{|V_{D}|\times K_{D}}\times R_{0+}^{|V_{S}|\times K_{S}},$$

 

(12) $$B=f_{w_{1}}(G,F)\in R_{0+}^{K_{D}\times K_{D}\times K_{S}},$$

where $g_{w_{0}}$ and $f_{w_{1}}$ are hypergraph neural networks based on message passing (Yadati, 2020) with parameters to learn *w*_0_, *w*_1_, $H^{d}=\{h^{d}(u)\in R_{0+}^{K_{D}}|u\in V_{D}\}$ (node representations of drugs) and $H^{s}=\{h^{s}(t)\in R_{0+}^{K_{S}}|t\in V_{S}\}$ (node representations of side effects). The formulation of each message passing layer has the following form:

(13) $$h^{\left(l+1\right)}(a)=\sigma \left(T\left({\left\{M^{\left(l\right)}\left(a,h^{\left(l\right)}(a),{\left\{\left(b,h^{\left(l\right)}(a)\right)\right\}}_{b\in e}\right)\right\}}_{e\in N_{a}}\right)\right),$$

where $h^{\left(l\right)}(a)$ is the representation of node $a\in V_{D}\cup V_{S}$ at layer (*l*), *σ* is an activation function, T is an aggregation function (e.g. an average function), $N_{a}=\{e\in E|a\in e\}$ and $M^{\left(l\right)}$ is a message passing function at layer (*l*) to pass information from neighbor nodes in hyperedge *e* to *a*:

(14) $$M^{\left(l\right)}\left(a,h^{\left(l\right)}(a),{\left\{\left(b,h^{\left(l\right)}(b)\right)\right\}}_{b\in e}\right)=$$

 

(15) $$\sum_{b\in e}M^{\left(l\right)}(c(a),c(b),h^{\left(l\right)}(a),h^{\left(l\right)}(b)),$$

where $M^{\left(l\right)}$ is a two-layer feedforward neural network, $c(b)=1\;\text{if}\;b\in V_{D}$ and $c(b)=-1\;\text{if}\;b\in V_{S}$ are the node types.

#### 3.3.3 Decoder

The reconstruction of the hypergraph is from the latent interaction assumption. The likelihood to reconstruct each triple $e=(u,v,t)\in V_{D}\times V_{D}\times V_{E}$ follows a Gaussian distribution:

(16) $$p(e|B,H)=\frac{1}{\sigma \sqrt{2\pi }}\text{exp}\;\left(-\frac{1}{2}{\left(\frac{i(e)-m_{w_{0},w_{1}}(e))}{\sigma }\right)}^{2}\right),$$

where i(e)=1 if e∈E, *i*(*e*) = 0 if $e\in \bar{E}=V_{D}\times V_{D}\times V_{S}/E$, and $m_{w_{0},w_{1}}(e)$ is the mean value for the latent interaction of *e*:

(17) $$m_{w_{0},w_{1}}(e)=B\times_{1}h^{d}(u)\times_{2}h^{d}(v)\times_{3}h^{s}(t).$$

Equation (17) is also the score for the interactions of triples (*u*, *v*, *t*) used for prediction. The likelihood for the decoder is:

(18) $$p(G|B,H)=\prod_{e=(u,v,t)\in V_{D}\times V_{D}\times V_{E}}p(e|B,H).$$

#### 3.3.4 Objective function

The objective function for our method is to maximize a posterior of the model. The objective function consists of two parts: one for log-likelihood of the model and one for the prior for sparsity control. Let $\Lambda \in R_{0+}^{K_{D}\times K_{D}\times K_{S}}$ be the horseshoe prior parameter for **B** and *τ* be the hyperparameter for the global sparsity of the horseshoe prior. We have the following objective function:

(19) $$\underset{B,H,\Lambda \geq 0}{\mathrm{argmax}}\underset{\text{log likelihood}}{\underset{︸}{\;\text{log}\;p(G|B,H)}}+\underset{\text{log of horseshoe prior}}{\underset{︸}{\;\text{log}\;p(B|\Lambda ,\tau )+\text{log}\;p(\Lambda )}},$$

where log p(G|B,H) is the log-likelihood of Equation (18) with H in Equation (11) and B in Equation (12), and log p(B|Λ,τ)+log p(Λ) is the logarithm of the horseshoe prior:

(20) $$\text{log}\;p(B|\Lambda ,\tau )=\sum \frac{-1}{2}{\left(\frac{B_{i,j,k}}{\tau \Lambda_{i,j,k}}\right)}^{2}+\sum \;\text{log}\;\Lambda_{i,j,k}^{-1}+\mathrm{const},$$

 

(21) $$\text{log}\;p(\Lambda )=\sum \;\text{log}\;\frac{1}{1+\Lambda_{i,k,j}^{2}}.$$

We then use stochastic gradient descent libraries in the PyTorch framework for optimizing Equation (19).

We also consider two other variants of SPARSE: ${\text{SPARSE}}_{O}$ for not using any sparsity prior and ${\text{SPARSE}}_{L}$ for using Laplace prior (Lasso regularization), to examine the effect of using the horseshoe prior.

## 4 Experimental results

We validated SPARSE in two scenarios: synthetic data and real data. On the synthetic data, assuming that the data are generated from the latent interactions, we examined if SPARSE can recover the latent interactions under changing hyperparameters of data: the number of latent features, sparsity and amount of noise. On real data, we checked the prediction performance of SPARSE in comparison with state-of-the-art DDI prediction methods by using three real-world DDI datasets. Additionally, we evaluated if the top unknown predictions by SPARSE can be related to biological phenomena like functions and mechanisms.

For all experiments, we used 20-fold cross-validation by dividing hyperedges into 20-folds, keeping the same number of hyperedges (side effects) in each fold. We reported the mean and standard deviation of the two commonly used measures AUC and AUPR. Also, all reported results were the highest performances through grid searches of hyperparameters. There were three hyperparameters for grid searches for SPARSE: (i) latent feature sizes. The tested values were 30, 40, 50 and 60. We set the same size for all layers. (ii) Global sparsity *τ*. The tested values were 0.01, 0.02, 0.03, 0.05 and 0.1 and (iii) the numbers of neural layers. The tested values were 1, 2 and 3. The hyperparameter values obtained were 50 for the latent feature size, τ=0.02 for TWOSIDES and τ=0.01 for CADDDI and JADERDDI, and the number of neural layers was 2. All experiments were run in a computer with Intel Core I7-9700 CPU, 8 GB GeForce RTX 2080 GPU and 32 GB RAM.

### 4.1 Synthetic data

#### 4.1.1 Data generation

The generation process for synthetic data consists of two steps: (i) generating latent interactions and (ii) generating triples of interacting drug–drug-side effects from the latent interactions, as follows.

1. Generating latent interactions. Given sets of indices of drug latent features: $L_{D}=\{1,2,\ldots ,K_{D}\}$ and side effect latent features: $L_{S}=\{1,2,\ldots ,K_{S}\}$.
  - Initialize a set of latent interactions A=∅.
  - For each $k\in L_{S}$:
    1. Sample the number of drug latent feature pairs: $n_{k}=\mathrm{RandomInteger}(M)$, where *M* is the maximum number of pairs.
    2. Sample *n_k_* pairs $(i,j)\in L_{D}\times L_{D}$. For each pair (*i*, *j*): A=A∪{(i,j,k)}.
2. Generating drug interactions:
  - Generate drug and side effect latent features. Assume that there are *V_D_* drugs and *V_S_* side effects.
    1. For each drug $u\in V_{D}$:
      - Sample the number of drug latent features: $n_{u}=\mathrm{RandomInter}(N_{1})$, where *N*_1_ is the maximum number of drug latent features.
      - Sample $g_{u}\subset L_{D},|g_{u}|=n_{u}$. For drug feature vectors *F*: $m_{u}\in R_{0+}^{K_{D}\times c},\;m_{u}\leftarrow 0,\;m_{u}[i]=1\;\text{if}\;\left\lfloor i/c\right\rfloor \in g_{u},\;f_{u}=\mathrm{Gaussian}(m_{u},\delta )$. $F=F\cup f_{u}$.
    2. For each side effect $t\in V_{S}$, sample the number of side effect latent feature $n_{t}=\mathrm{RandomInter}(N_{2})$ and Sample $g_{t}\subset L_{S},|g_{t}|=n_{t}$.
  - Generating true triples $E^{*}$. Initialize $E^{*}\to \emptyset$. For $(u,v,t)\in V_{D}\times V_{D}\times V_{S}$, if $g_{u}\times g_{v}\times g_{t}\cap A!=\emptyset$ then (*u*, *v*, *t*) is a true triple: $E^{*}=E^{*}\cup (u,v,t)$.
  - Adding noise:
    1. For each $e\in E^{*}$, replace *e* by a random sample $e\prime \in \bar{E^{*}}=V_{D}\times V_{D}\times V_{S}/E^{*}$ with probability *r*. The final set of triples of drug–drug-side effects is *E*.

Finally, we have a synthetic dataset with triples of drug–drug-side effects *E* and drug feature vectors *F*.

#### 4.1.2 Experiments

The synthetic data has five hyperparameters: the number of drugs, the number of side effects, the number of latent interactions, data sparsity and the amount of noise (noise rate). We evaluated our methods by changing one hyperparameter, fixing the other four. The hyperparameters changed are (i) number of latent features, (ii) data sparsity and (iii) noise rate.

##### 1) Changing the number of latent features

*Setting*: $V_{D}=400,V_{S}=300$, noise rate *r *=* *0.01. We changed $K_{D}=K_{S}\in \{5,10,20,30,40,50\}$. For each (*K_D_*, *K_S_*), we selected *N*_1_, *N*_2_ and *M* such that the sparsity of the generated data is kept at 0.98.

*Compared methods*: We compared four methods: ${\text{SPARSE}}_{O}$ (no sparsity control), CentSmoothie (Nguyen *et al.*, 2021), a similarity-based hypergraph neural network, HPNN (Feng *et al.*, 2019) and SBM on hypergraph (Anandkumar *et al.*, 2013).

**Fig. 2.**
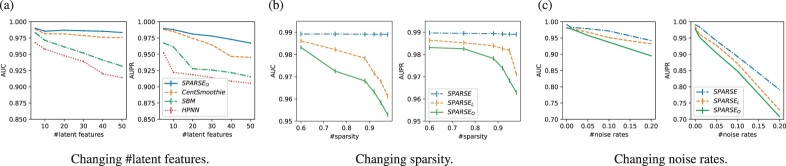
Performances on synthetic data, when changing (**a**) #latent features, (**b**) sparsity and (**c**) amount of noise

*Results:*  Figure 2a shows the results, where ${\text{SPARSE}}_{O}$ achieved the highest performances among the compared methods in all cases. We had the following two findings:

1. For the small number of latent features, the performance of CentSmoothie was close to ${\text{SPARSE}}_{O}$ (both AUC and AUPR were around 0.99 under $K_{D}=K_{S}=5$). However, by increasing the number of latent features, the performance gap between ${\text{SPARSE}}_{O}$ and CentSmoothie also increased (gaps in AUC and AUPR were around 0.01 and 0.03, respectively, when $K_{D}=K_{S}=50$). This result implies that CentSmoothie was unable to distinguish latent interactions clearly for a large number of latent interactions, while ${\text{SPARSE}}_{O}$ worked better for capturing multiple latent interactions.
2. The performances of SBM were lower than both CentSmoothie and ${\text{SPARSE}}_{O}$, since SBM did not use the node features, which decreased the performance. HPNN, a similarity-based hypergraph neural network, had the lowest performance since the two drugs of a DDI do not necessarily have similarity in the data generated from latent interactions. Overall, these results indicated that ${\text{SPARSE}}_{O}$ can recover the latent interactions better than the other methods.

##### 2) Changing data sparsity

*Setting*: $V_{D}=400,V_{S}=300,\;K_{D}=K_{S}=50,\;N_{1}=N_{2}=4$ and r=0.01. We changed *M* in {50,40,30,20,10,5}, resulting in data sparsity in {0.6,0.75,0.88,0.92,0.95,0.98}, respectively.

*Compared methods*: Since in the previous experiment, ${\text{SPARSE}}_{O}$ outperformed the compared methods already, we compared SPARSE and two variants ${\text{SPARSE}}_{L}$ and ${\text{SPARSE}}_{O}$ (please see the end of Section 3.3.4) to check the effect of sparse priors.

*Results*: Figure 2b shows the results, where SPARSE achieved the highest performance, followed by ${\text{SPARSE}}_{L}$ and ${\text{SPARSE}}_{O}$. In particular, the performance advantage by SPARSE using sparsity control was clearer with higher sparsity. These results indicate that the horseshoe prior is suitable for learning sparse data.

##### 3) Changing the amount of noise

*Setting*: $V_{D}=400,V_{S}=300,\;K_{D}=K_{S}=50,\;N_{1}=N_{2}=4$, *M *=* *1 (keeping the data sparsity of 0.98). We changed noise *r* in [0,0.01,0.05,0.10,0.20].

*Compared methods*: We again compared SPARSE with two variants ${\text{SPARSE}}_{L}$ and ${\text{SPARSE}}_{O}$ to examine the effectiveness of the sparse priors to deal with noise.

*Results:*  Figure 2c shows the results, where again SPARSE achieved the highest performances among the three methods for all different amounts of noise. When there are no noises, the performances of the three methods were very close to each other. However, as the amount of noise is increased, the advantage of SPARSE over the other two methods became clearer. For example, when the amount of noise is 20%, the gap between SPARSE and ${\text{SPARSE}}_{L}$ reached around 0.07, and the gap between SPARSE and ${\text{SPARSE}}_{O}$ was around 0.1. These results suggest that the horseshoe prior could deal with noise better than the Laplace prior and the case with no sparsity prior.

### 4.2 Real data

#### 4.2.1 Data description

We used three real-world datasets for DDI, namely TWOSIDES (Tatonetti *et al.*, 2012), CADDDI and JADERDDI. To our knowledge, TWOSIDES is the largest benchmark dataset for DDI. The other two datasets, i.e. CADDDI and JADERDDI, were generated from Canada Vigilance Adverse Reaction Reports and Japanese Adverse Drug Event Reports, respectively, in the same manner as the way that TWOSIDES was generated from the adverse events reported to US Food and Drug Administration (Nguyen *et al.*, 2021). For all datasets, we only chose small molecular drugs, which can be found in DrugBank. Also, we focused drugs appearing in more than five interactions (hyperedges) in each dataset. For each drug, we used a feature (binary) vector, with the size of 2329, consisting of 881 substructures and 1448 interacting proteins. Table 1 shows a summary statistics of the three real benchmark datasets, TWOSIDES, CADDDI and JADERDDI.

#### 4.2.2 Predictive performance experiments

*Compared methods:* For our method, we used SPARSE and two variants ${\text{SPARSE}}_{O}$ and ${\text{SPARSE}}_{L}$. We further used five methods as competing methods against SPARSE. These competing methods were CentSmoothie (Nguyen *et al.*, 2021), the traditional similarity-based hypergraph neural network (HPNN) (Feng *et al.*, 2019), two DDI graph-based graph neural networks: Decagon (Zitnik *et al.*, 2018) and SpecConv (Kipf and Welling, 2016) and, a molecular graph-based graph neural network, MRGNN (Xu *et al.*, 2019). Decagon and CentSmoothie provide available codes, and we ran them with the recommended settings. For MLNN, MGRNN, SpecConv, HPNN and SBM, we implemented them and did a grid search for finding the best hyperparameter values.

*Results—Cross-validation predictive performance:*  Table 2 shows AUC and AUPR results of all competing methods. From this table, SPARSE and two variants (${\text{SPARSE}}_{L}$ and ${\text{SPARSE}}_{O}$) achieved the highest performances, followed by CentSmoothie, SBM and HPNN. On the other hand, the performances of SpecConv, Decagon and MRGNN were significantly lower. Amazingly, ${\text{SPARSE}}_{O}$ (SPARSE without any sparsity prior) achieved still better performance over CentSmoothie, particularly in AUPR. There was only one case (CADDDI), where the AUC of SPARSE was slightly smaller than that of CentSmoothie. We then ran *t*-test over the prediction results of these two methods, to examine the significance of the difference between CentSmoothie and SPARSE. The resultant *P*-value of *t*-test was 0.057, indicating that the performance advantage of CentSmoothie over SPARSE was NOT significant, under the regular significance level of 0.05. Also, it has to be noted that AUPR is more useful than AUC for imbalanced data (Saito and Rehmsmeier, 2015), which can be often seen practically. We emphasize that DDI is a typical example of this situation. In fact, the AUPR performance gap between ${\text{SPARSE}}_{O}$ and CentSmoothie reached around 1%, 5% and 12% in TWOSIDES, CADDDI and JADERDDI, respectively. The performance gap in JADERDDI is especially sizable. This might be caused by the high sparsity of JADERDDI (see Table 1).

**Table 2.** Comparison of performances of the methods on the real DDI datasets

Method	TWOSIDES		CADDDI		JADERDDI	
	AUC	AUPR	AUC	AUPR	AUC	AUPR
MRGNN	0.8452 ± 0.0036	0.8029 ± 0.0039	0.9226 ± 0.0015	0.7113 ± 0.0031	0.9049 ± 0.0009	0.3698 ± 0.0019
Decagon	0.8639 ± 0.0029	0.8094 ± 0.0024	0.9132 ± 0.0014	0.6338 ± 0.0029	0.9099 ± 0.0012	0.4710 ± 0.0027
SpecConv	0.8785 ± 0.0025	0.8256 ± 0.0022	0.8971 ± 0.0055	0.6640 ± 0.0014	0.8862 ± 0.0025	0.5162 ± 0.0047
HPNN	0.9044 ± 0.0003	0.8410 ± 0.0007	0.9495 ± 0.0004	0.7020 ± 0.0018	0.9127 ± 0.0004	0.5198 ± 0.0016
SBM	0.9337 ± 0.0002	0.8583 ± 0.0004	0.9588 ± 0.0006	0.8170 ± 0.0008	0.9428 ± 0.0006	0.5963 ± 0.0018
CentSmoothie	0.9348 ± 0.0002	0.8749 ± 0.0013	0.9846 ± 0.0001	0.8230 ± 0.0019	0.9684 ± 0.0004	0.6044 ± 0.0025
${\text{SPARSE}}_{O}$	0.9511 ± 0.0002	0.8811 ± 0.0001	0.9824 ± 0.0009	0.8773 ± 0.0014	0.9692 ± 0.0007	0.7230 ± 0.0008
${\text{SPARSE}}_{L}$	0.9517 ± 0.0001	0.8815 ± 0.0002	**0.9859 **±** **0.0007	0.8797 ± 0.0010	0.9694 ± 0.0011	0.7276 ± 0.0017
SPARSE	**0.9524 **±** **0.0001	**0.8820 **±** **0.0002	0.9837 ± 0.0010	**0.8843 **±** **0.0012	**0.9698 **±** **0.0008	**0.7348 **±** **0.0018

These results suggest that the latent interaction assumption in SPARSE is more reasonable and suitable for DDI prediction than CentSmoothie and the other competing methods. Among SPARSE, ${\text{SPARSE}}_{L}$ and ${\text{SPARSE}}_{O}$, SPARSE achieved the highest performance. Note that the performance gap between SPARSE and ${\text{SPARSE}}_{L}$ in AUPR became clearer for more sparse data: e.g. only around 0.1% for TWOSIDES, while the gap reached around 1% for CADDDI and JADERDDI. Hence, we can see that with more sparse data, the horseshoe prior had advantage over Laplace prior and also the case with no sparsity prior.

*Results—Unknown DDI prediction performance:* We evaluated the predictive ability of unknown DDIs. That is, we first trained a model by using the whole TWOSIDES data (the largest dataset), then predicted the scores of unknown triples (drug–drug-side effect), and finally sorted the predicted triples in the descending order of the scores. We focused on the top 400 predictions of each method and checked the overlap with the DDIs stored in drugs.com (Drugs.com, 2021; Thelwall *et al.*, 2017), a commonly used web checker for DDIs. Table 3 shows the number of overlaps between the DDIs in drugs.com and the top 400 predictions. SPARSE found 98 overlapped DDIs with drugs.com, this number being the highest and followed by CentSmoothie with 71 and HPNN with 48.

**Table 3.** Number of overlaps with DDIs in drugs.com for the top 400 predictions

Method	#Overlaps
SPARSE	98
CentSmoothie	71
HPNN	48

#### 4.2.3 Case studies: interpretation of top 10 unknown predictions

SPARSE is an SBM with latent features for drugs, side effects, and interactions. In particular, the model has connections between latent drug features and latent interactions. Thus from the trained model, we can extract the drug features, which are most associated with each drug latent feature and further extract the drug features most associated with each latent interaction through the corresponding latent drug feature. This means that we can retrieve drug features of a DDI if we can connect the DDI with the latent interactions. Algorithm 1 shows the pseudocode of this procedure (with *T* = 20 in our cases). SPARSE is a sparse model, which allows only a limited number of latent interactions and eventually allows to extract only a limited number of drug features. This is a sizable advantage of SPARSE for understanding the biological/chemical background behind predicted DDIs.

Algorithm 1 Extracting potentially associated drug features

**Input:** Learned parameters $B\in R_{0+}^{K_{D}\times K_{D}\times K_{S}},\;H^{d}=\{h^{d}(u)\}\in R_{0+}^{|V_{D}|\times K_{D}},\;H^{s}=\{h^{s}(u)\}\in R_{0+}^{|V_{S}|\times K_{S}}$, drug features matrix $F^{d}=\{f^{d}(u)\}\in R_{0+}^{|V_{D}|\times K_{O}}$, a predicted triple (*u*, *v*, *t*), hyperparameter *T*

**Outout:** Associated drug features for the triple

* //Extract drug features for each latent feature*

* ***for**  $k\in 1\ldots K_{D}$  **do**

*  *$a_{k}=\{j|\text{Correlation}(H_{.,k}^{d},F_{.,j}^{d})\text{in top}\;T\}$

* ***end for**

*//Calculate non-zeros latent interactions.* ⊙  *is the pairwise dot product*,

* ⊗ is the outer product.*

* *
 

$$\begin{array}{c} ss=B⊙(h^{d}(u)⊗h^{d}(v)⊗h^{s}(t)\\ tt=\{(i,j,k)|ss_{i,j,k}>0\} \end{array}$$

* //Extract potentially associated drug features for the triple*

* *Re←∅

* ***for**  (i,j,k)∈tt  **do**

$Re\leftarrow Re\cup \{(\text{Non-zero features of}\;f^{d}(u)\in a_{i},\;\text{Non-zero features of}\;f^{d}(v)\in a_{k})\}$

* ***end for**

* *Return *Re*

For case studies, we extracted drug features (such as protein/pathway names) of the top unknown DDI predictions by using SPARSE, which was trained by the entire TWOSIDES. Table 4 shows the top 10 predictions (out of the 400 predictions in the experiment of the previous section) with the observable features associated with latent drug features [fifth column from the right-hand side. In this column, ‘*Not clear*’ means that to our current understanding of the potential DDI mechanisms, we could not explain the corresponding low-level (molecular level) background, although our algorithm could find associated drug features], the target protein of the corresponding drug using DrugBank (sixth column) and the corresponding reference to each DDI (seventh column). The top predictions are likely to be similar to each other, since the similar triples are likely to have similar scores. In fact the top predictions in Table 4 have large overlaps, but from the table, we could find the following four points:

**Table 4.** Top 10 new (unknown) predictions with potentially associated latent features of proteins and extracted proteins

No.	Drug A	Drug B	Side effect	Observable features associated with latent features	Extracted proteins of drugs from DrugBank	References
1	Ciprofloxacin	Mefenamic acid	Abdominal distension	Cytochrome enzymes	—	Venkataraman *et al.* (2014)
2	Naratriptan	Oxycodone	Abnormal ECG	Serotonin transporters and receptors	—	Baldo (2018) and Ritter *et al.* (2019)
3	Naratriptan	Tramadol	Abnormal ECG	Serotonin transporters and receptors	—	Baldo (2018)
4	Naratriptan	Sertraline	Abnormal ECG	Serotonin transporters and receptors	5-Hydroxytryptamine receptor 1B (and 1D) and sodium-dependent serotonin transporter	Ritter *et al.* (2019)
5	Naratriptan	Paroxetine	Abnormal ECG	Serotonin transporters and receptors	5-Hydroxytryptamine receptor 1B (and 1D) and sodium-dependent serotonin transporter	Ritter *et al.* (2019)
6	Trihexyphenidyl	Thiothixene	Abnormal EEG	Dopamine receptors	—	Ritter *et al.* (2019)
7	Carisoprodol	Orphenadrine	Abnormal vision	Not clear	—	Downs *et al.* (2019)
8	Buspirone	Orphenadrine	Abnormal vision	Not clear	—	Ritter *et al.* (2019)
9	Oxycodone	Orphenadrine	Abnormal vision	Not clear	—	Ritter *et al.* (2019)
10	Carisoprodol	Zaleplon	Abnormal vision	Not clear	—	Fagiolini *et al.* (2004)

1. The fourth and fifth predictions show the cases, where SPARSE could specify target proteins precisely, confirming the high credibility of these predictions and more importantly, approving the high ability of SPARSE for detecting unknown DDIs.
2. The first, second, third and sixth predictions show the cases, where SPARSE could identify possible interacting protein groups (fourth column), not necessarily directly associated with the drugs, indicating that SPARSE allows suggesting novel interactions as well as potential target proteins.
3. The validity of the seventh, eighth, ninth and tenth predictions might be understood by high-level views, like the connection between vision and dizziness/sedation. This result implies that SPARSE can predict probable interactions, which however cannot be straightforwardly inferred from low-level data.
4. Entirely, we could find relevant references for all top 10 predictions (Baldo, 2018; Fagiolini *et al.*, 2004; Rho *et al.*, 1997; Venkataraman *et al.*, 2014), giving plausibility of these prediction and at the same time an additional layer of evidence for the usefulness of SPARSE in practical settings. To facilitate medical research and confirmation of our findings by subsequent clinical or preclinical studies, we provide the potential mechanisms as a Supplementary Material for our top predictions. Also, we discuss below the main biological mechanism for a predicted top 10 interaction:
*Naratriptan, Sertraline and abnormal ECG*: Sertraline belongs to the selective serotonin reuptake inhibitor class antidepressants. Members of this class inhibit the reuptake of the neurotransmitter serotonin into cells (Ritter *et al.*, 2019). Through this inhibition, sertraline increases serotonin levels outside of the cells and allows serotonin to remain longer at its site of action. Naratriptan is known to cause heart-related side effects through serotonin receptor agonism at serotonin type 1 receptors (Dodick *et al.*, 2004; Ritter *et al.*, 2019). Therefore, the predicted side effect can be a direct consequence of sertraline increasing the level of endogenous serotonin and naratriptan acting at serotonin receptors in the heart, with the resulting changes visible in electrocardiogram recordings.

## 5 Conclusion and discussion

We have proposed SPARSE to learn the latent representations of drugs, side effects and interactions, through hypergraph neural networks. SPARSE addresses three important issues of state-of-the-art DDI prediction, which have not been addressed by any other methods. Extensive empirical validation using both synthetic and real data showed that SPARSE outperformed all current, cutting-edge methods for DDI prediction, verifying the effectiveness of multiple types of latent interaction assumptions and the sparsity control setting of SPARSE.

Possible future work is to generalize SPARSE for higher-order drug interactions with multiple drugs. Another interesting direction might be to apply SPARSE to other sparse, high-dimensional data in bioinformatics.

## Funding

This work was supported in part by Otsuka Toshimi Scholarship Foundation (to D.A.N.); MEXT KAKENHI [grant number 22K12150] (to C.H.N.); the Japan Society for the Promotion of Science (Postdoctoral Fellowships for Research in Japan, standard program) [P20809] (to P.P.); and MEXT KAKENHI [grant numbers: 19H04169, 20F20809, 21H05027 and 22H03645] and the AIPSE program of the Academy of Finland (to H.M.).

*Conflict of Interest*: none declared.

## Supplementary Material

btac250_Supplementary_Data
