# Supplementary material for: SPARSE: a sparse hypergraph neural network for learning multiple types of latent combinations to accurately predict drug–drug interactions
Source: Bioinformatics. 2022 Jun 27;38(Suppl 1):i333–41. doi: 10.1093/bioinformatics/btac250 (PMC9235485; doi:10.1093/bioinformatics/btac250)
Supplement: btac250_Supplementary_Data [file btac250_supplementary_data.pdf]

Supplemental materials

1 Illustrations of learned latent interactions on synthetic data.

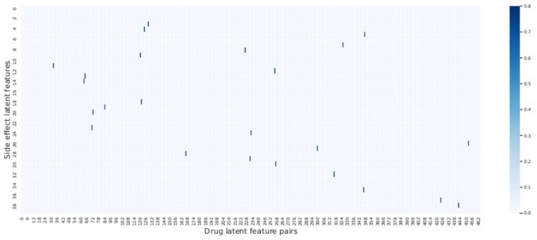

(a) True latent interactions

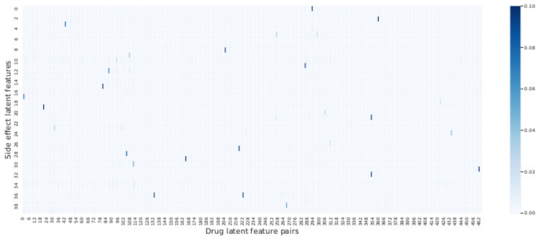

(b) SPARSE

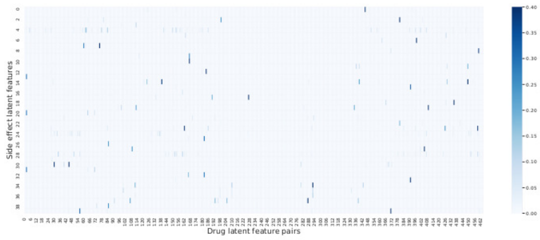

(c) $\mathrm{SPARSE_L}$

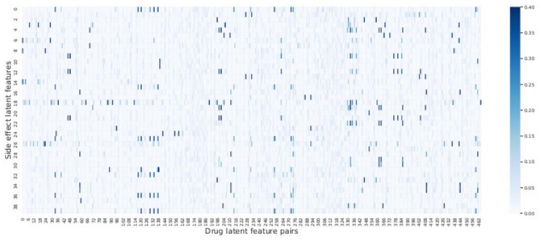

(d) $\mathrm{SPARSE_O}$

Figure 1: Illustrations of learned latent interactions of SPARSE (and variants) on synthetic data.

We visualized the learned latent interactions on the synthetic data in Fig. 1, where the x-axis is for pairs of drug latent features, the y-axis is for the side effect latent feature, and the latent interactions are shown as dots. We used the data with the sparsity of 0.98.

Fig. 1 (a) is the true latent interactions which were used to generate sparse (only a few number of) DDIs. Fig. 1 (b), 1 (c), and 1 (d) are learned latent interactions of SPARSE, SPARSE_L, and SPARSE_O, respectively. We can see that the learned latent interactions of SPARSE were the closest one to the true latent interactions (Fig. 1 (a)). On the other hand, the other methods captured non-significant interactions also. Hence, these results show that SPARSE with the horseshoe prior was suitable to deal with sparse data.

2 Sensitivity of SPARSE by changing the global sparsity hyperparameter τ

We examined the sensitivity of SPARSE by changing global sparsity hyperparameter τ in (10^{-10}, 10^{-5}, 0.001, 0.01, 0.02, 0.03, 0.05, 0.1, 0.5 ,1, 10, 100, 1000, 10^5 and 10^{10}). The results are in Fig. 2. The x-axis is $\log_{10}$ of τ, and the y-axis is AUPR.

We can see that SPARSE achieved the highest performance with $\log_{10}(\tau)$ of around -3 and 0 (τ from 0.001 to 1) and decreased as $\tau \to 0$ or $\tau \to \infty$. This is a reasonable, expected result, since as $\tau \to 0$, the horseshoe regularization term becomes stronger and as $\tau \to \infty$, the horseshoe regularization term becomes weaker (and eventually no regularization).

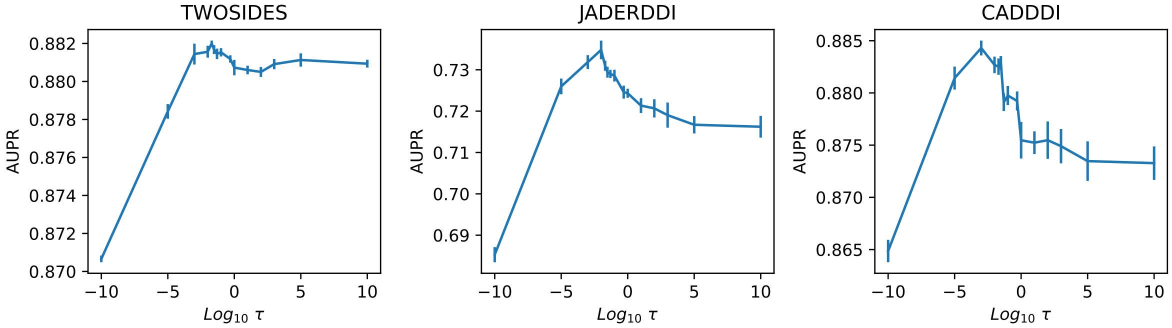

Figure 2: Sensitivity of SPARSE by changing the global sparsity hyperparameter τ.

3 Top 98 interactions predicted from SPARSE

- The following content shows the 98 top predictions from SPARSE.
- Each prediction is a triple of drug-drug-side effect. The potentially associated proteins (from DrugBank) and pathways (from KEGG) for interactions of the drugs are extracted from SPARSE.
- The explanation with expert knowledge for the first 10 predicted interactions is provided.

1. **Predicted interaction: Ciprofloxacin, Mefenamic acid, abdominal distension**
   - For drug Ciprofloxacin:
     - Proteins: Cytochrome P450 3A4,DNA gyrase subunit A,DNA topoisomerase 4 subunit A
     - Pathways: Retinol metabolism - Homo sapiens (human),Drug metabolism - cytochrome P450 - Homo sapiens (human),Steroid hormone biosynthesis - Homo sapiens (human),Linoleic acid metabolism - Homo sapiens (human)
   - For drug Mefenamic acid:
     - Proteins: Cytochrome P450 2C8,Prostaglandin G/H synthase 1,Prostaglandin G/H synthase 2
     - Pathways: Human papillomavirus infection - Homo sapiens (human),Kaposi sarcoma-associated herpesvirus infection - Homo sapiens (human),Human cytomegalovirus infection - Homo sapiens (human)
   - Explanation: Mefenamic acid, a non-steroidal antiinflammatory drug, is metabolized into many active metabolites through the hepatic enzyme CYP1A2. The active metabolites might be responsible for acute hepatotoxicity cases after mefenamic acid use (Venkataraman et al., 2014, Chem Res Toxicol). Ciprofloxacin is a well-know inhibitor of CYP1A2 and thereby, it might shift active metabolite formation of mefenamic acid. This shift in the composition of active metabolites may increase the chance of hepatotoxicity, of which a prominent symptom is abdominal distension.

2. **Predicted interaction: Naratriptan, Oxycodone, abnormal ecg**
   - For drug Naratriptan:
     - Proteins: 5-hydroxytryptamine receptor 1A,Amine oxidase [flavin-containing] A,5-hydroxytryptamine receptor 1D,5-hydroxytryptamine receptor 1B,5-hydroxytryptamine receptor 1F
     - Pathways: Dopaminergic synapse - Homo sapiens (human),Tyrosine metabolism - Homo sapiens (human),Alcoholism - Homo sapiens (human),Cocaine addiction - Homo sapiens (human),Amphetamine addiction - Homo sapiens (human),Tryptophan metabolism - Homo sapiens (human),Glycine, serine and threonine metabolism - Homo sapiens (human),Phenylalanine metabolism - Homo sapiens (human),Histidine metabolism - Homo sapiens (human),Arginine and proline metabolism - Homo sapiens (human)

- For drug Oxycodone:
  - Proteins: Alpha-1-acid glycoprotein 1,Mu-type opioid receptor,Delta-type opioid receptor,Kappa-type opioid receptor
  - Pathways: Sphingolipid signaling pathway - Homo sapiens (human)
- Explanation: Opioids, like oxycodone, are known to cause, albeit relatively rare, serotonin syndrome (Baldo, 2018, Arch Toxicol). While the exact mechanism remains unknown, elevated levels of serotonin have been observed after opioid class analgesics. Naratriptan is a known agonist of particular serotonin receptors (5-HT type 1 receptors), including those in the heart. Through this mechanism a rare side effect of naratriptan is chest pain, related to the alteration of coronary circulation. An abnormal electrocardiography (ECG) signal might be the consequence of concomittant activation of heart serotonergic receptors, when already elevated serotonin levels are present.

3. **Predicted interaction: Naratriptan, Tramadol, abnormal ecg**
   - For drug Naratriptan:
     - Proteins: 5-hydroxytryptamine receptor 1A,Amine oxidase [flavin-containing] A,5-hydroxytryptamine receptor 1D,5-hydroxytryptamine receptor 1B,5-hydroxytryptamine receptor 1F
     - Pathways: Dopaminergic synapse - Homo sapiens (human),Tyrosine metabolism - Homo sapiens (human),Alcoholism - Homo sapiens (human),Cocaine addiction - Homo sapiens (human),Amphetamine addiction - Homo sapiens (human),Tryptophan metabolism - Homo sapiens (human),Glycine, serine and threonine metabolism - Homo sapiens (human),Phenylalanine metabolism - Homo sapiens (human),Histidine metabolism - Homo sapiens (human),Arginine and proline metabolism - Homo sapiens (human)
   - For drug Tramadol:
     - Proteins: Cytochrome P450 2B6,Sodium-dependent serotonin transporter,Mu-type opioid receptor,Delta-type opioid receptor,Kappa-type opioid receptor,Alpha-7 nicotinic cholinergic receptor subunit
     - Pathways: Sphingolipid signaling pathway - Homo sapiens (human),Serotonergic synapse - Homo sapiens (human),Arachidonic acid metabolism - Homo sapiens (human)
   - Explanation: Opioids, like tramadol, are known to cause serotonin syndrome (Baldo, 2018, Arch Toxicol), with elevated levels of serotonin after the use of opioid class analgesics. Naratriptan causes changes in coronary circulation via serotonin type 1 receptors in the heart. Therefore, an abnormal ECG signal might be the consequence of concomittant activation of heart serotonergic receptors, when already elevated serotonin levels are present.

4. **Predicted interaction: Naratriptan, Sertraline, abnormal ecg**
   - For drug Naratriptan:
     - Proteins: 5-hydroxytryptamine receptor 1A,Amine oxidase [flavin-containing] A,5-hydroxytryptamine receptor 1D,5-hydroxytryptamine receptor 1B,5-hydroxytryptamine receptor 1F
     - Pathways: Dopaminergic synapse - Homo sapiens (human),Tyrosine metabolism - Homo sapiens (human),Alcoholism - Homo sapiens (human),Cocaine addiction - Homo sapiens (human),Amphetamine addiction - Homo sapiens (human),Tryptophan metabolism - Homo sapiens (human),Glycine, serine and threonine metabolism - Homo sapiens (human),Phenylalanine metabolism - Homo sapiens (human),Histidine metabolism - Homo sapiens (human),Arginine and proline metabolism - Homo sapiens (human)
   - For drug Sertraline:
     - Proteins: Membrane-associated progesterone receptor component 1,Cytochrome P450 2B6,Sodium-dependent serotonin transporter,CYP2B protein
     - Pathways: Serotonergic synapse - Homo sapiens (human),Arachidonic acid metabolism - Homo sapiens (human)
   - Explanation: Sertraline belongs to the selective serotonin reuptake inhibitors that inhibit the reuptake of serotonin into neurons, thereby increasing serotonin levels. Naratriptan is known

to cause heart related side effects through serotonin receptor agonism at serotonin type 1 receptors. Therefore, the predicted side effect can be a direct consequence of naratriptan acting at serotonin receptors, and sertraline directly increasing the level of endogenous serotonin.

5. **Predicted interaction: Naratriptan, Paroxetine, abnormal ecg**
   - For drug Naratriptan:
     - Proteins: 5-hydroxytryptamine receptor 1A,Amine oxidase [flavin-containing] A,5-hydroxytryptamine receptor 1D,5-hydroxytryptamine receptor 1B,5-hydroxytryptamine receptor 1F
     - Pathways: Dopaminergic synapse - Homo sapiens (human),Tyrosine metabolism - Homo sapiens (human),Alcoholism - Homo sapiens (human),Cocaine addiction - Homo sapiens (human),Amphetamine addiction - Homo sapiens (human),Tryptophan metabolism - Homo sapiens (human),Glycine, serine and threonine metabolism - Homo sapiens (human),Phenylalanine metabolism - Homo sapiens (human),Histidine metabolism - Homo sapiens (human),Arginine and proline metabolism - Homo sapiens (human)
   - For drug Paroxetine:
     - Proteins: Cytochrome P450 2B6,Sodium-dependent serotonin transporter
     - Pathways: Serotonergic synapse - Homo sapiens (human),Arachidonic acid metabolism - Homo sapiens (human)
   - Explanation: Paroxetine, similarly to sertraline, also belongs to the selective serotonin reuptake inhibitors. Therefore, like in the previous case, paroxetine can increase serotonin levels, while naratriptan can activate serotonin type 1 receptors in the heart. This combined synergism might cause the an abnormal ECG signal clinically.
6. **Predicted interaction: Trihexyphenidyl, Thiothixene, abnormal eeg**
   - For drug Trihexyphenidyl:
     - Proteins: Muscarinic acetylcholine receptor M2,Muscarinic acetylcholine receptor M4,Muscarinic acetylcholine receptor M5,Muscarinic acetylcholine receptor M1,Muscarinic acetylcholine receptor M3
     - Pathways: Calcium signaling pathway - Homo sapiens (human),Regulation of actin cytoskeleton - Homo sapiens (human)
   - For drug Thiothixene:
     - Proteins: D(2) dopamine receptor,D(1A) dopamine receptor,5-hydroxytryptamine receptor 2A
     - Pathways: Gap junction - Homo sapiens (human),Dopaminergic synapse - Homo sapiens (human),Rap1 signaling pathway - Homo sapiens (human),Calcium signaling pathway - Homo sapiens (human),Inflammatory mediator regulation of TRP channels - Homo sapiens (human),Alcoholism - Homo sapiens (human),Parkinson disease - Homo sapiens (human),Cocaine addiction - Homo sapiens (human),Amphetamine addiction - Homo sapiens (human)
   - Explanation: Thiothixene belongs to the class of atypical antipsychotics and acts at dopamine receptors (among others). While trihexyphenidyl is a drug known for its effects at muscarinic acetylcholine receptors, based on in vitro data it may also cause dopamine release (Downs et al., 2019, Neurobiol Dis). Since dopamine is a major neurotransmitter within the brain, changes in its levels and the parallel interference with its receptors might cause altered electroencephalography (EEG) signals.
7. **Predicted interaction: Carisoprodol, Orphenadrine, abnormal vision**
   - For drug Carisoprodol:
     - Proteins: Cytochrome P450 2C19
     - Pathways: Chemical carcinogenesis - Homo sapiens (human),Drug metabolism - cytochrome P450 - Homo sapiens (human),Serotonergic synapse - Homo sapiens (human),Arachidonic acid metabolism - Homo sapiens (human),Linoleic acid metabolism - Homo sapiens (human)

- For drug Orphenadrine:
  - Proteins: Glutamate receptor ionotropic, NMDA 2D,Glutamate receptor ionotropic, NMDA 3B,Cytochrome P450 1A2,Cytochrome P450 3A4,Cytochrome P450 2B6,Sodium-dependent noradrenaline transporter,Histamine H1 receptor,Glutamate receptor ionotropic, NMDA 1,Glutamate receptor ionotropic, NMDA 3A,Sodium channel protein type 10 subunit alpha
  - Pathways: Nicotine addiction - Homo sapiens (human),Ras signaling pathway - Homo sapiens (human),Glutamatergic synapse - Homo sapiens (human),Circadian entrainment - Homo sapiens (human),Amyotrophic lateral sclerosis (ALS) - Homo sapiens (human),Alzheimer disease - Homo sapiens (human),Huntington disease - Homo sapiens (human),Long-term potentiation - Homo sapiens (human)
- Explanation: Carisoprodol is a drug used for musculoskeletal pain and causes sedation and dizziness. Orphenadrine is a drug that can also cause dizziness, sedation and sleepiness. A general notion about drugs that can cause dizziness is that they can cause vision problems mainly by changing visual perception. Therefore, the synergistic effects of these drugs on vigilance may explain the predicted side effect.

8. **Predicted interaction: Buspirone, Orphenadrine, abnormal vision**

- For drug Buspirone:
  - Proteins: 5-hydroxytryptamine receptor 1A,Cytochrome P450 2D6,D(2) dopamine receptor,Cytochrome P450 3A5,Cytochrome P450 3A7
  - Pathways: Chemical carcinogenesis - Homo sapiens (human),Drug metabolism - cytochrome P450 - Homo sapiens (human),Endocrine resistance - Homo sapiens (human),Taste transduction - Homo sapiens (human),Serotonergic synapse - Homo sapiens (human)
- For drug Orphenadrine:
  - Proteins: Glutamate receptor ionotropic, NMDA 2D,Glutamate receptor ionotropic, NMDA 3B,Cytochrome P450 1A2,Cytochrome P450 3A4,Cytochrome P450 2B6,Sodium-dependent noradrenaline transporter,Histamine H1 receptor,Glutamate receptor ionotropic, NMDA 1,Glutamate receptor ionotropic, NMDA 3A,Sodium channel protein type 10 subunit alpha
  - Pathways: Nicotine addiction - Homo sapiens (human),Ras signaling pathway - Homo sapiens (human),Glutamatergic synapse - Homo sapiens (human),Circadian entrainment - Homo sapiens (human),Amyotrophic lateral sclerosis (ALS) - Homo sapiens (human),Alzheimer disease - Homo sapiens (human),Huntington disease - Homo sapiens (human),Long-term potentiation - Homo sapiens (human)
- Explanation: Buspirone is a drug used for long-term treatment of anxiety and may also cause dizziness. Dizziness might cause vision problems and altered perception of visual stimuli, therefore together with orphenadrine that can cause a similar side effect (dizziness), they can potentiate abnormal visual perception.

9. **Predicted interaction: Oxycodone, Orphenadrine, abnormal vision**

- For drug Oxycodone:
  - Proteins: Alpha-1-acid glycoprotein 1,Cytochrome P450 2D6,Cytochrome P450 3A5,Cytochrome P450 3A7
  - Pathways: Chemical carcinogenesis - Homo sapiens (human),Drug metabolism - cytochrome P450 - Homo sapiens (human),Endocrine resistance - Homo sapiens (human),Serotonergic synapse - Homo sapiens (human)
- For drug Orphenadrine:
  - Proteins: Glutamate receptor ionotropic, NMDA 2D,Glutamate receptor ionotropic, NMDA 3B,Cytochrome P450 1A2,Cytochrome P450 3A4,Cytochrome P450 2B6,Sodium-dependent noradrenaline transporter,Histamine H1 receptor,Glutamate receptor ionotropic, NMDA 1,Glutamate receptor ionotropic, NMDA 3A,Sodium channel protein type 10 subunit alpha

  - Pathways: Nicotine addiction - Homo sapiens (human),Ras signaling pathway - Homo sapiens (human),Glutamatergic synapse - Homo sapiens (human),Circadian entrainment - Homo sapiens (human),Amyotrophic lateral sclerosis (ALS) - Homo sapiens (human),Alzheimer disease - Homo sapiens (human),Huntington disease - Homo sapiens (human),Long-term potentiation - Homo sapiens (human)
- Explanation: Oxycodone is an opioid analgesic, known to cause sedation and might also interfere with vision directly through constriction of the pupils. Orphenadrine is an anti-cholinergic drug that can cause the dilation of pupils. Therefore, the combination of these drugs can cause abnormal vision, depending on their net effects. In addition to direct effects on pupils, the common adverse effect caused by both drugs is dizziness, which may further contribute to abnormal vision.

10. **Predicted interaction: Carisoprodol, Zaleplon, abnormal vision**
    - For drug Carisoprodol:
      - Proteins: Cytochrome P450 2C19
      - Pathways: Chemical carcinogenesis - Homo sapiens (human),Drug metabolism - cytochrome P450 - Homo sapiens (human),Serotonergic synapse - Homo sapiens (human),Arachidonic acid metabolism - Homo sapiens (human),Linoleic acid metabolism - Homo sapiens (human)
    - For drug Zaleplon:
      - Proteins: Cytochrome P450 3A4,Gamma-aminobutyric acid receptor subunit alpha-1,Cytochrome P450 3A7,Translocator protein,Aldehyde oxidase
      - Pathways: Nicotine addiction - Homo sapiens (human),Nicotinate and nicotinamide metabolism - Homo sapiens (human),Vitamin B6 metabolism - Homo sapiens (human)
    - Explanation: Carisoprodol, in addition to its primary unknown mechanism of action, is metabolized to meprobamate in the human body. Meprobamate can bind to GABA-A receptors (Rho et al., 1997, J Pharmacol Exp Ther). Zaleplon is a drug well-known to exert its effects through the same receptor. Therefore, combination of the two can result in excessive effects, like sedation, dizziness and consequent vision changes. At the same time, modulation of GABA-A has been demonstrated to be important in postnatal visual cortex development, (Fagiolini et al., 2004, Science), thus, even more direct effects on vision may be possible.

11. **Predicted interaction: Propranolol, Orphenadrine, abnormal vision**
    - For drug Propranolol:
      - Proteins: 5-hydroxytryptamine receptor 1A,Cytochrome P450 2D6,Cytochrome P450 3A5,Cytochrome P450 3A7,Cytochrome P450 2C19
      - Pathways: Chemical carcinogenesis - Homo sapiens (human),Drug metabolism - cytochrome P450 - Homo sapiens (human),Endocrine resistance - Homo sapiens (human),Taste transduction - Homo sapiens (human),Serotonergic synapse - Homo sapiens (human),Arachidonic acid metabolism - Homo sapiens (human),Linoleic acid metabolism - Homo sapiens (human)
    - For drug Orphenadrine:
      - Proteins: Glutamate receptor ionotropic, NMDA 2D,Glutamate receptor ionotropic, NMDA 3B,Cytochrome P450 1A2,Cytochrome P450 3A4,Cytochrome P450 2B6,Sodium-dependent noradrenaline transporter,Histamine H1 receptor,Glutamate receptor ionotropic, NMDA 1,Glutamate receptor ionotropic, NMDA 3A,Sodium channel protein type 10 subunit alpha
      - Pathways: Nicotine addiction - Homo sapiens (human),Ras signaling pathway - Homo sapiens (human),Glutamatergic synapse - Homo sapiens (human),Circadian entrainment - Homo sapiens (human),Amyotrophic lateral sclerosis (ALS) - Homo sapiens (human),Alzheimer disease - Homo sapiens (human),Huntington disease - Homo sapiens (human),Long-term potentiation - Homo sapiens (human)

12. **Predicted interaction: Buspirone, Zaleplon, abnormal vision**

- For drug Buspirone:
  - Proteins: 5-hydroxytryptamine receptor 1A,Cytochrome P450 2D6,D(2) dopamine receptor,Cytochrome P450 3A5,Cytochrome P450 3A7
  - Pathways: Chemical carcinogenesis - Homo sapiens (human),Drug metabolism - cytochrome P450 - Homo sapiens (human),Endocrine resistance - Homo sapiens (human),Taste transduction - Homo sapiens (human),Serotonergic synapse - Homo sapiens (human)
- For drug Zaleplon:
  - Proteins: Cytochrome P450 3A4,Gamma-aminobutyric acid receptor subunit alpha-1,Cytochrome P450 3A7,Translocator protein,Aldehyde oxidase
  - Pathways: Nicotine addiction - Homo sapiens (human),Nicotinate and nicotinamide metabolism - Homo sapiens (human),Vitamin B6 metabolism - Homo sapiens (human)

13. **Predicted interaction: Doxazosin, Orphenadrine, abnormal vision**
    - For drug Doxazosin:
      - Proteins: Cytochrome P450 2D6,Cytochrome P450 2C19
      - Pathways: Chemical carcinogenesis - Homo sapiens (human),Drug metabolism - cytochrome P450 - Homo sapiens (human),Endocrine resistance - Homo sapiens (human),Serotonergic synapse - Homo sapiens (human),Arachidonic acid metabolism - Homo sapiens (human),Linoleic acid metabolism - Homo sapiens (human)
    - For drug Orphenadrine:
      - Proteins: Glutamate receptor ionotropic, NMDA 2D,Glutamate receptor ionotropic, NMDA 3B,Cytochrome P450 1A2,Cytochrome P450 3A4,Cytochrome P450 2B6,Sodium-dependent noradrenaline transporter,Histamine H1 receptor,Glutamate receptor ionotropic, NMDA 1,Glutamate receptor ionotropic, NMDA 3A,Sodium channel protein type 10 subunit alpha
      - Pathways: Nicotine addiction - Homo sapiens (human),Ras signaling pathway - Homo sapiens (human),Glutamatergic synapse - Homo sapiens (human),Circadian entrainment - Homo sapiens (human),Amyotrophic lateral sclerosis (ALS) - Homo sapiens (human),Alzheimer disease - Homo sapiens (human),Huntington disease - Homo sapiens (human),Long-term potentiation - Homo sapiens (human)
14. **Predicted interaction: Diltiazem, Orphenadrine, abnormal vision**
    - For drug Diltiazem:
      - Proteins: Cytochrome P450 2D6,Cytochrome P450 3A5,Cytochrome P450 3A7,Cytochrome P450 2C19
      - Pathways: Chemical carcinogenesis - Homo sapiens (human),Drug metabolism - cytochrome P450 - Homo sapiens (human),Endocrine resistance - Homo sapiens (human),Serotonergic synapse - Homo sapiens (human),Arachidonic acid metabolism - Homo sapiens (human),Linoleic acid metabolism - Homo sapiens (human)
    - For drug Orphenadrine:
      - Proteins: Glutamate receptor ionotropic, NMDA 2D,Glutamate receptor ionotropic, NMDA 3B,Cytochrome P450 1A2,Cytochrome P450 3A4,Cytochrome P450 2B6,Sodium-dependent noradrenaline transporter,Histamine H1 receptor,Glutamate receptor ionotropic, NMDA 1,Glutamate receptor ionotropic, NMDA 3A,Sodium channel protein type 10 subunit alpha
      - Pathways: Nicotine addiction - Homo sapiens (human),Ras signaling pathway - Homo sapiens (human),Glutamatergic synapse - Homo sapiens (human),Circadian entrainment - Homo sapiens (human),Amyotrophic lateral sclerosis (ALS) - Homo sapiens (human),Alzheimer disease - Homo sapiens (human),Huntington disease - Homo sapiens (human),Long-term potentiation - Homo sapiens (human)
15. **Predicted interaction: Oxaprozin, Risedronate, acute myeloblastic leukemia**
    - For drug Oxaprozin:

- Proteins: Cytochrome P450 2C9,Prostaglandin G/H synthase 1,Prostaglandin G/H synthase 2
- Pathways: Small cell lung cancer - Homo sapiens (human),Human papillomavirus infection - Homo sapiens (human),Kaposi sarcoma-associated herpesvirus infection - Homo sapiens (human),C-type lectin receptor signaling pathway - Homo sapiens (human),TNF signaling pathway - Homo sapiens (human),Human cytomegalovirus infection - Homo sapiens (human),VEGF signaling pathway - Homo sapiens (human),Leishmaniasis - Homo sapiens (human),IL-17 signaling pathway - Homo sapiens (human),NF-kappa B signaling pathway - Homo sapiens (human)

• For drug Risedronate:
  - Proteins: Farnesyl pyrophosphate synthase,Prostaglandin G/H synthase 2
  - Pathways: Small cell lung cancer - Homo sapiens (human),C-type lectin receptor signaling pathway - Homo sapiens (human),Human T-cell leukemia virus 1 infection - Homo sapiens (human),TNF signaling pathway - Homo sapiens (human),VEGF signaling pathway - Homo sapiens (human),Influenza A - Homo sapiens (human),Terpenoid backbone biosynthesis - Homo sapiens (human),Leishmaniasis - Homo sapiens (human),IL-17 signaling pathway - Homo sapiens (human),NF-kappa B signaling pathway - Homo sapiens (human)

16. **Predicted interaction: Pamidronate, Etodolac, adenitis**

• For drug Pamidronate:
  - Proteins: Farnesyl pyrophosphate synthase
  - Pathways: Human T-cell leukemia virus 1 infection - Homo sapiens (human),Influenza A - Homo sapiens (human),Terpenoid backbone biosynthesis - Homo sapiens (human)

• For drug Etodolac:
  - Proteins: Cytochrome P450 2C9,UDP-glucuronosyltransferase 2B7,Prostaglandin G/H synthase 2
  - Pathways: Chemical carcinogenesis - Homo sapiens (human),Drug metabolism - cytochrome P450 - Homo sapiens (human),MicroRNAs in cancer - Homo sapiens (human),Serotonergic synapse - Homo sapiens (human),Arachidonic acid metabolism - Homo sapiens (human),Linoleic acid metabolism - Homo sapiens (human)

17. **Predicted interaction: Nadolol, Bupropion, agitated**

• For drug Nadolol:
  - Proteins: Beta-2 adrenergic receptor,Beta-1 adrenergic receptor
  - Pathways: Unknown

• For drug Bupropion:
  - Proteins: Neuronal acetylcholine receptor subunit alpha-3,Sodium-dependent dopamine transporter
  - Pathways: Neuroactive ligand-receptor interaction - Homo sapiens (human)

18. **Predicted interaction: Orphenadrine, Cyclobenzaprine, allergic dermatitis**

• For drug Orphenadrine:
  - Proteins: Glutamate receptor ionotropic, NMDA 2D,Glutamate receptor ionotropic, NMDA 3B,Cytochrome P450 1A2,Cytochrome P450 3A4,Cytochrome P450 2B6,Sodium-dependent noradrenaline transporter,Histamine H1 receptor,Glutamate receptor ionotropic, NMDA 1,Glutamate receptor ionotropic, NMDA 3A,Sodium channel protein type 10 subunit alpha
  - Pathways: Nicotine addiction - Homo sapiens (human),Ras signaling pathway - Homo sapiens (human),Glutamatergic synapse - Homo sapiens (human),Circadian entrainment - Homo sapiens (human),Amyotrophic lateral sclerosis (ALS) - Homo sapiens (human),Alzheimer disease - Homo sapiens (human),Huntington disease - Homo sapiens (human),Long-term potentiation - Homo sapiens (human)

- For drug Cyclobenzaprine:
  - Proteins: 5-hydroxytryptamine receptor 2A
  - Pathways: Inflammatory mediator regulation of TRP channels - Homo sapiens (human)

19. **Predicted interaction: Zaleplon, Fentanyl, allergic dermatitis**
    - For drug Zaleplon:
      - Proteins: Cytochrome P450 3A4,Gamma-aminobutyric acid receptor subunit alpha-1,Cytochrome P450 3A7,Translocator protein,Aldehyde oxidase
      - Pathways: Nicotine addiction - Homo sapiens (human),Nicotinate and nicotinamide metabolism - Homo sapiens (human),Vitamin B6 metabolism - Homo sapiens (human)
    - For drug Fentanyl:
      - Proteins:
      - Pathways: Unknown
20. **Predicted interaction: Zaleplon, Mirtazapine, allergic dermatitis**
    - For drug Zaleplon:
      - Proteins: Cytochrome P450 3A4,Gamma-aminobutyric acid receptor subunit alpha-1,Cytochrome P450 3A7,Translocator protein,Aldehyde oxidase
      - Pathways: Nicotine addiction - Homo sapiens (human),Nicotinate and nicotinamide metabolism - Homo sapiens (human),Vitamin B6 metabolism - Homo sapiens (human)
    - For drug Mirtazapine:
      - Proteins: D(2) dopamine receptor,5-hydroxytryptamine receptor 2A,Sodium-dependent serotonin transporter,Histamine H1 receptor,Histamine H3 receptor
      - Pathways: Dopaminergic synapse - Homo sapiens (human),Rap1 signaling pathway - Homo sapiens (human),Inflammatory mediator regulation of TRP channels - Homo sapiens (human),Alcoholism - Homo sapiens (human),Parkinson disease - Homo sapiens (human),Cocaine addiction - Homo sapiens (human)
21. **Predicted interaction: Zaleplon, Olanzapine, allergic dermatitis**
    - For drug Zaleplon:
      - Proteins: Cytochrome P450 3A4,Gamma-aminobutyric acid receptor subunit alpha-1,Cytochrome P450 3A7,Translocator protein,Aldehyde oxidase
      - Pathways: Nicotine addiction - Homo sapiens (human),Nicotinate and nicotinamide metabolism - Homo sapiens (human),Vitamin B6 metabolism - Homo sapiens (human)
    - For drug Olanzapine:
      - Proteins: Muscarinic acetylcholine receptor M5,D(2) dopamine receptor,Histamine H2 receptor,5-hydroxytryptamine receptor 2A,Histamine H1 receptor,5-hydroxytryptamine receptor 5A
      - Pathways: Dopaminergic synapse - Homo sapiens (human),Rap1 signaling pathway - Homo sapiens (human),Inflammatory mediator regulation of TRP channels - Homo sapiens (human),Alcoholism - Homo sapiens (human),Parkinson disease - Homo sapiens (human),Cocaine addiction - Homo sapiens (human)
22. **Predicted interaction: Zaleplon, Venlafaxine, angiitis**
    - For drug Zaleplon:
      - Proteins: Cytochrome P450 3A4,Gamma-aminobutyric acid receptor subunit alpha-1,Cytochrome P450 3A7,Translocator protein,Aldehyde oxidase
      - Pathways: Nicotine addiction - Homo sapiens (human),Nicotinate and nicotinamide metabolism - Homo sapiens (human),Vitamin B6 metabolism - Homo sapiens (human)
    - For drug Venlafaxine:
      - Proteins: Sodium-dependent serotonin transporter

    - Pathways: Unknown

23. **Predicted interaction: Zaleplon, Sertraline, angiitis**
    - For drug Zaleplon:
        - Proteins: Cytochrome P450 3A4,Gamma-aminobutyric acid receptor subunit alpha-1,Cytochrome P450 3A7,Translocator protein,Aldehyde oxidase
        - Pathways: Nicotine addiction - Homo sapiens (human),Nicotinate and nicotinamide metabolism - Homo sapiens (human),Vitamin B6 metabolism - Homo sapiens (human)
    - For drug Sertraline:
        - Proteins: Membrane-associated progesterone receptor component 1,Sodium-dependent serotonin transporter
        - Pathways: Unknown
24. **Predicted interaction: Orphenadrine, Pregabalin, aphonia**
    - For drug Orphenadrine:
        - Proteins: Glutamate receptor ionotropic, NMDA 2D,Glutamate receptor ionotropic, NMDA 3B,Cytochrome P450 1A2,Cytochrome P450 3A4,Cytochrome P450 2B6,Sodium-dependent noradrenaline transporter,Histamine H1 receptor,Glutamate receptor ionotropic, NMDA 1,Glutamate receptor ionotropic, NMDA 3A,Sodium channel protein type 10 subunit alpha
        - Pathways: Nicotine addiction - Homo sapiens (human),Ras signaling pathway - Homo sapiens (human),Glutamatergic synapse - Homo sapiens (human),Circadian entrainment - Homo sapiens (human),Amyotrophic lateral sclerosis (ALS) - Homo sapiens (human),Alzheimer disease - Homo sapiens (human),Huntington disease - Homo sapiens (human),Long-term potentiation - Homo sapiens (human)
    - For drug Pregabalin:
        - Proteins: Voltage-dependent P/Q-type calcium channel subunit alpha-1A,Excitatory amino acid transporter 3
        - Pathways: Dopaminergic synapse - Homo sapiens (human)
25. **Predicted interaction: Orphenadrine, Mirtazapine, aphonia**
    - For drug Orphenadrine:
        - Proteins: Glutamate receptor ionotropic, NMDA 2D,Glutamate receptor ionotropic, NMDA 3B,Cytochrome P450 1A2,Cytochrome P450 3A4,Cytochrome P450 2B6,Sodium-dependent noradrenaline transporter,Histamine H1 receptor,Glutamate receptor ionotropic, NMDA 1,Glutamate receptor ionotropic, NMDA 3A,Sodium channel protein type 10 subunit alpha
        - Pathways: Nicotine addiction - Homo sapiens (human),Ras signaling pathway - Homo sapiens (human),Glutamatergic synapse - Homo sapiens (human),Circadian entrainment - Homo sapiens (human),Amyotrophic lateral sclerosis (ALS) - Homo sapiens (human),Alzheimer disease - Homo sapiens (human),Huntington disease - Homo sapiens (human),Long-term potentiation - Homo sapiens (human)
    - For drug Mirtazapine:
        - Proteins: D(2) dopamine receptor,5-hydroxytryptamine receptor 2A,Sodium-dependent serotonin transporter,Histamine H1 receptor,Histamine H3 receptor
        - Pathways: Dopaminergic synapse - Homo sapiens (human),Rap1 signaling pathway - Homo sapiens (human),Inflammatory mediator regulation of TRP channels - Homo sapiens (human),Alcoholism - Homo sapiens (human),Parkinson disease - Homo sapiens (human),Cocaine addiction - Homo sapiens (human)
26. **Predicted interaction: Orphenadrine, Promethazine, aphonia**
    - For drug Orphenadrine:

- Proteins: Glutamate receptor ionotropic, NMDA 2D,Glutamate receptor ionotropic, NMDA 3B,Cytochrome P450 1A2,Cytochrome P450 3A4,Cytochrome P450 2B6,Sodium-dependent noradrenaline transporter,Histamine H1 receptor,Glutamate receptor ionotropic, NMDA 1,Glutamate receptor ionotropic, NMDA 3A,Sodium channel protein type 10 subunit alpha
  - Pathways: Nicotine addiction - Homo sapiens (human),Ras signaling pathway - Homo sapiens (human),Glutamatergic synapse - Homo sapiens (human),Circadian entrainment - Homo sapiens (human),Amyotrophic lateral sclerosis (ALS) - Homo sapiens (human),Alzheimer disease - Homo sapiens (human),Huntington disease - Homo sapiens (human),Long-term potentiation - Homo sapiens (human)
- For drug Promethazine:
  - Proteins: Muscarinic acetylcholine receptor M5,D(2) dopamine receptor,Histamine H2 receptor,5-hydroxytryptamine receptor 2A,Histamine H1 receptor
  - Pathways: Dopaminergic synapse - Homo sapiens (human),Rap1 signaling pathway - Homo sapiens (human),Inflammatory mediator regulation of TRP channels - Homo sapiens (human),Alcoholism - Homo sapiens (human),Parkinson disease - Homo sapiens (human),Cocaine addiction - Homo sapiens (human)

27. **Predicted interaction: Orphenadrine, Fentanyl, aphonia**
    - For drug Orphenadrine:
      - Proteins: Glutamate receptor ionotropic, NMDA 2D,Glutamate receptor ionotropic, NMDA 3B,Cytochrome P450 1A2,Cytochrome P450 3A4,Cytochrome P450 2B6,Sodium-dependent noradrenaline transporter,Histamine H1 receptor,Glutamate receptor ionotropic, NMDA 1,Glutamate receptor ionotropic, NMDA 3A,Sodium channel protein type 10 subunit alpha
      - Pathways: Nicotine addiction - Homo sapiens (human),Ras signaling pathway - Homo sapiens (human),Glutamatergic synapse - Homo sapiens (human),Circadian entrainment - Homo sapiens (human),Amyotrophic lateral sclerosis (ALS) - Homo sapiens (human),Alzheimer disease - Homo sapiens (human),Huntington disease - Homo sapiens (human),Long-term potentiation - Homo sapiens (human)
    - For drug Fentanyl:
      - Proteins:
      - Pathways: Unknown

28. **Predicted interaction: Orphenadrine, Venlafaxine, aphonia**
    - For drug Orphenadrine:
      - Proteins: Glutamate receptor ionotropic, NMDA 2D,Glutamate receptor ionotropic, NMDA 3B,Cytochrome P450 1A2,Cytochrome P450 3A4,Cytochrome P450 2B6,Sodium-dependent noradrenaline transporter,Histamine H1 receptor,Glutamate receptor ionotropic, NMDA 1,Glutamate receptor ionotropic, NMDA 3A,Sodium channel protein type 10 subunit alpha
      - Pathways: Nicotine addiction - Homo sapiens (human),Ras signaling pathway - Homo sapiens (human),Glutamatergic synapse - Homo sapiens (human),Circadian entrainment - Homo sapiens (human),Amyotrophic lateral sclerosis (ALS) - Homo sapiens (human),Alzheimer disease - Homo sapiens (human),Huntington disease - Homo sapiens (human),Long-term potentiation - Homo sapiens (human)
    - For drug Venlafaxine:
      - Proteins: Sodium-dependent serotonin transporter
      - Pathways: Unknown

29. **Predicted interaction: Orphenadrine, Olanzapine, aphonia**
    - For drug Orphenadrine:

- Proteins: Glutamate receptor ionotropic, NMDA 2D,Glutamate receptor ionotropic, NMDA 3B,Cytochrome P450 1A2,Cytochrome P450 3A4,Cytochrome P450 2B6,Sodium-dependent noradrenaline transporter,Histamine H1 receptor,Glutamate receptor ionotropic, NMDA 1,Glutamate receptor ionotropic, NMDA 3A,Sodium channel protein type 10 subunit alpha
  - Pathways: Nicotine addiction - Homo sapiens (human),Ras signaling pathway - Homo sapiens (human),Glutamatergic synapse - Homo sapiens (human),Circadian entrainment - Homo sapiens (human),Amyotrophic lateral sclerosis (ALS) - Homo sapiens (human),Alzheimer disease - Homo sapiens (human),Huntington disease - Homo sapiens (human),Long-term potentiation - Homo sapiens (human)
- For drug Olanzapine:
  - Proteins: Muscarinic acetylcholine receptor M5,D(2) dopamine receptor,Histamine H2 receptor,5-hydroxytryptamine receptor 2A,Histamine H1 receptor,5-hydroxytryptamine receptor 5A
  - Pathways: Dopaminergic synapse - Homo sapiens (human),Rap1 signaling pathway - Homo sapiens (human),Inflammatory mediator regulation of TRP channels - Homo sapiens (human),Alcoholism - Homo sapiens (human),Parkinson disease - Homo sapiens (human),Cocaine addiction - Homo sapiens (human)

30. **Predicted interaction: Clindamycin, Erythromycin, apoplexy**
    - For drug Clindamycin:
      - Proteins: 50S ribosomal protein L10
      - Pathways: Unknown
    - For drug Erythromycin:
      - Proteins: Motilin receptor,Albumin
      - Pathways: Unknown

31. **Predicted interaction: Cimetidine, Felodipine, arrhythmia**
    - For drug Cimetidine:
      - Proteins: Solute carrier family 22 member 3,Cytochrome P450 11B1, mitochondrial,Cytochrome P450 3A7,Histamine H2 receptor,Dimethylaniline monooxygenase [N-oxide-forming] 3,Dimethylaniline monooxygenase [N-oxide-forming] 1,Multidrug and toxin extrusion protein 1,Solute carrier family 22 member 4
      - Pathways: Cortisol synthesis and secretion - Homo sapiens (human),Cushing syndrome - Homo sapiens (human),Gastric acid secretion - Homo sapiens (human)
    - For drug Felodipine:
      - Proteins: Troponin C, skeletal muscle,Calcium/calmodulin-dependent 3',5'-cyclic nucleotide phosphodiesterase 1A,Troponin C, slow skeletal and cardiac muscles,Calcium/calmodulin-dependent 3',5'-cyclic nucleotide phosphodiesterase 1B
      - Pathways: Olfactory transduction - Homo sapiens (human)

32. **Predicted interaction: Nortriptyline, Oxycodone, aspartate aminotransferase increase**
    - For drug Nortriptyline:
      - Proteins: Membrane-associated progesterone receptor component 1,5-hydroxytryptamine receptor 2C,Alpha-2A adrenergic receptor,Prostaglandin G/H synthase 1,5-hydroxytryptamine receptor 6
      - Pathways: Platelet activation - Homo sapiens (human)
    - For drug Oxycodone:
      - Proteins: Alpha-1-acid glycoprotein 1,Cytochrome P450 3A4,Cytochrome P450 2D6,Cytochrome P450 3A7
      - Pathways: Unknown

33. **Predicted interaction: Haloperidol, Scopolamine, aspergillosis**

- For drug Haloperidol:
  - Proteins: Carbonyl reductase [NADPH] 1
  - Pathways: Unknown
- For drug Scopolamine:
  - Proteins: Sucrase-isomaltase, intestinal
  - Pathways: Carbohydrate digestion and absorption - Homo sapiens (human),Galactose metabolism - Homo sapiens (human),Starch and sucrose metabolism - Homo sapiens (human)

34. **Predicted interaction: Levetiracetam, Scopolamine, aspergillosis**

- For drug Levetiracetam:
  - Proteins: Synaptic vesicle glycoprotein 2A
  - Pathways: ECM-receptor interaction - Homo sapiens (human)
- For drug Scopolamine:
  - Proteins: Sucrase-isomaltase, intestinal
  - Pathways: Carbohydrate digestion and absorption - Homo sapiens (human),Galactose metabolism - Homo sapiens (human),Starch and sucrose metabolism - Homo sapiens (human)

35. **Predicted interaction: Pramipexole, Carisoprodol, aspiration pneumonia**

- For drug Pramipexole:
  - Proteins:
  - Pathways: Unknown
- For drug Carisoprodol:
  - Proteins: Cytochrome P450 2C19
  - Pathways: Chemical carcinogenesis - Homo sapiens (human),Drug metabolism - cytochrome P450 - Homo sapiens (human),Serotonergic synapse - Homo sapiens (human),Arachidonic acid metabolism - Homo sapiens (human),Linoleic acid metabolism - Homo sapiens (human)

36. **Predicted interaction: Nefazodone, Carisoprodol, aspiration pneumonia**

- For drug Nefazodone:
  - Proteins: CYP2B protein
  - Pathways: Unknown
- For drug Carisoprodol:
  - Proteins: Cytochrome P450 2C19
  - Pathways: Chemical carcinogenesis - Homo sapiens (human),Drug metabolism - cytochrome P450 - Homo sapiens (human),Serotonergic synapse - Homo sapiens (human),Arachidonic acid metabolism - Homo sapiens (human),Linoleic acid metabolism - Homo sapiens (human)

37. **Predicted interaction: Orphenadrine, Nortriptyline, atherosclerosis**

- For drug Orphenadrine:
  - Proteins: Glutamate receptor ionotropic, NMDA 2D,Glutamate receptor ionotropic, NMDA 3B,Cytochrome P450 1A2,Cytochrome P450 3A4,Cytochrome P450 2B6,Sodium-dependent noradrenaline transporter,Histamine H1 receptor,Glutamate receptor ionotropic, NMDA 1,Glutamate receptor ionotropic, NMDA 3A,Sodium channel protein type 10 subunit alpha

  - Pathways: Nicotine addiction - Homo sapiens (human),Ras signaling pathway - Homo sapiens (human),Glutamatergic synapse - Homo sapiens (human),Circadian entrainment - Homo sapiens (human),Amyotrophic lateral sclerosis (ALS) - Homo sapiens (human),Alzheimer disease - Homo sapiens (human),Huntington disease - Homo sapiens (human),Long-term potentiation - Homo sapiens (human)

- For drug Nortriptyline:
  - Proteins: Membrane-associated progesterone receptor component 1,5-hydroxytryptamine receptor 1A,5-hydroxytryptamine receptor 2C,Cytochrome P450 2D6,D(2) dopamine receptor,Cytochrome P450 3A5,Cytochrome P450 2C19
  - Pathways: Chemical carcinogenesis - Homo sapiens (human),Drug metabolism - cytochrome P450 - Homo sapiens (human),Endocrine resistance - Homo sapiens (human),Taste transduction - Homo sapiens (human),Serotonergic synapse - Homo sapiens (human),Arachidonic acid metabolism - Homo sapiens (human),Linoleic acid metabolism - Homo sapiens (human)

38. **Predicted interaction: Zaleplon, Oxycodone, atherosclerosis**

- For drug Zaleplon:
  - Proteins: Cytochrome P450 3A4,Gamma-aminobutyric acid receptor subunit alpha-1,Cytochrome P450 3A7,Translocator protein,Aldehyde oxidase
  - Pathways: Nicotine addiction - Homo sapiens (human),Nicotinate and nicotinamide metabolism - Homo sapiens (human),Vitamin B6 metabolism - Homo sapiens (human)
- For drug Oxycodone:
  - Proteins: Alpha-1-acid glycoprotein 1,Cytochrome P450 2D6,Cytochrome P450 3A5,Cytochrome P450 3A7
  - Pathways: Chemical carcinogenesis - Homo sapiens (human),Drug metabolism - cytochrome P450 - Homo sapiens (human),Endocrine resistance - Homo sapiens (human),Serotonergic synapse - Homo sapiens (human)

39. **Predicted interaction: Oxaprozin, Sulfasalazine, autonomic instability**

- For drug Oxaprozin:
  - Proteins: Prostaglandin G/H synthase 1,Prostaglandin G/H synthase 2
  - Pathways: Small cell lung cancer - Homo sapiens (human),VEGF signaling pathway - Homo sapiens (human),Leishmaniasis - Homo sapiens (human),IL-17 signaling pathway - Homo sapiens (human),NF-kappa B signaling pathway - Homo sapiens (human)
- For drug Sulfasalazine:
  - Proteins: Inhibitor of nuclear factor kappa-B kinase subunit beta,Inhibitor of nuclear factor kappa-B kinase subunit alpha,Phospholipase A2,Polyunsaturated fatty acid 5-lipoxygenase,Prostaglandin G/H synthase 1,Thromboxane-A synthase,Acetyl-CoA acetyltransferase, mitochondrial,Prostaglandin G/H synthase 2,Peroxisome proliferator-activated receptor gamma,Proton-coupled folate transporter,Cystine/glutamate transporter
  - Pathways: Small cell lung cancer - Homo sapiens (human),VEGF signaling pathway - Homo sapiens (human),Glyoxylate and dicarboxylate metabolism - Homo sapiens (human),Butanoate metabolism - Homo sapiens (human),Propanoate metabolism - Homo sapiens (human),Leishmaniasis - Homo sapiens (human),IL-17 signaling pathway - Homo sapiens (human),NF-kappa B signaling pathway - Homo sapiens (human),Fatty acid metabolism - Homo sapiens (human),Synthesis and degradation of ketone bodies - Homo sapiens (human)

40. **Predicted interaction: Cimetidine, Naproxen, back ache**

- For drug Cimetidine:
  - Proteins: Solute carrier family 22 member 3,Histamine H2 receptor,Dimethylaniline monooxygenase [N-oxide-forming] 1,Multidrug and toxin extrusion protein 1

  - Pathways: Neuroactive ligand-receptor interaction - Homo sapiens (human)
- For drug Naproxen:
  - Proteins:
  - Pathways: Unknown

41. **Predicted interaction: Zaleplon, Tramadol, back ache**
    - For drug Zaleplon:
      - Proteins:
      - Pathways: Unknown
    - For drug Tramadol:
      - Proteins: Cytochrome P450 3A4,Sodium-dependent noradrenaline transporter,Alpha-7 nicotinic cholinergic receptor subunit
      - Pathways: Chemical carcinogenesis - Homo sapiens (human),Retinol metabolism - Homo sapiens (human),Metabolism of xenobiotics by cytochrome P450 - Homo sapiens (human),Drug metabolism - cytochrome P450 - Homo sapiens (human),Bile secretion - Homo sapiens (human),Linoleic acid metabolism - Homo sapiens (human)
42. **Predicted interaction: Carbamazepine, Nortriptyline, basal cell carcinoma**
    - For drug Carbamazepine:
      - Proteins: Cytochrome P450 3A4,UDP-glucuronosyltransferase 2B7,Cytochrome P450 3A7,RalA-binding protein 1
      - Pathways: Unknown
    - For drug Nortriptyline:
      - Proteins: Membrane-associated progesterone receptor component 1,5-hydroxytryptamine receptor 2C,Alpha-2A adrenergic receptor,Prostaglandin G/H synthase 1,5-hydroxytryptamine receptor 6
      - Pathways: Platelet activation - Homo sapiens (human)
43. **Predicted interaction: Sumatriptan, Mirtazapine, biliary tract disorder**
    - For drug Sumatriptan:
      - Proteins: 5-hydroxytryptamine receptor 1A,Amine oxidase [flavin-containing] A,5-hydroxytryptamine receptor 1D,5-hydroxytryptamine receptor 1B,5-hydroxytryptamine receptor 1F,Solute carrier organic anion transporter family member 1A2,Broad substrate specificity ATP-binding cassette transporter ABCG2,Solute carrier organic anion transporter family member 1B1
      - Pathways: Tyrosine metabolism - Homo sapiens (human),Tryptophan metabolism - Homo sapiens (human),Glycine, serine and threonine metabolism - Homo sapiens (human),Phenylalanine metabolism - Homo sapiens (human),Histidine metabolism - Homo sapiens (human),Arginine and proline metabolism - Homo sapiens (human)
    - For drug Mirtazapine:
      - Proteins: Alpha-2A adrenergic receptor,D(2) dopamine receptor,Alpha-2C adrenergic receptor,D(1A) dopamine receptor,5-hydroxytryptamine receptor 2A,5-hydroxytryptamine receptor 2C,5-hydroxytryptamine receptor 7,Alpha-1A adrenergic receptor,Histamine H1 receptor,D(3) dopamine receptor,5-hydroxytryptamine receptor 2B,5-hydroxytryptamine receptor 3A,Sodium-dependent dopamine transporter,Histamine H3 receptor
      - Pathways: Gap junction - Homo sapiens (human),AMPK signaling pathway - Homo sapiens (human),Adrenergic signaling in cardiomyocytes - Homo sapiens (human),Dopaminergic synapse - Homo sapiens (human),Rap1 signaling pathway - Homo sapiens (human),Salivary secretion - Homo sapiens (human),Inflammatory mediator regulation of TRP channels - Homo sapiens (human),Alcoholism - Homo sapiens (human),Parkinson disease - Homo sapiens (human),Cocaine addiction - Homo sapiens (human)

44. **Predicted interaction: Mirtazapine, Rizatriptan, biliary tract disorder**

- For drug Mirtazapine:
  - Proteins: Alpha-2A adrenergic receptor,D(2) dopamine receptor,Alpha-2C adrenergic receptor,D(1A) dopamine receptor,5-hydroxytryptamine receptor 2A,5-hydroxytryptamine receptor 2C,5-hydroxytryptamine receptor 7,Alpha-1A adrenergic receptor,Histamine H1 receptor,D(3) dopamine receptor,5-hydroxytryptamine receptor 2B,5-hydroxytryptamine receptor 3A,Sodium-dependent dopamine transporter,Histamine H3 receptor
  - Pathways: Gap junction - Homo sapiens (human),AMPK signaling pathway - Homo sapiens (human),Adrenergic signaling in cardiomyocytes - Homo sapiens (human),Dopaminergic synapse - Homo sapiens (human),Rap1 signaling pathway - Homo sapiens (human),Salivary secretion - Homo sapiens (human),Inflammatory mediator regulation of TRP channels - Homo sapiens (human),Alcoholism - Homo sapiens (human),Parkinson disease - Homo sapiens (human),Cocaine addiction - Homo sapiens (human)
- For drug Rizatriptan:
  - Proteins: Cytochrome P450 1A2,Amine oxidase [flavin-containing] A,5-hydroxytryptamine receptor 1D,5-hydroxytryptamine receptor 1B,5-hydroxytryptamine receptor 1F
  - Pathways: Taste transduction - Homo sapiens (human),Tyrosine metabolism - Homo sapiens (human),Alcoholism - Homo sapiens (human),Cocaine addiction - Homo sapiens (human),Amphetamine addiction - Homo sapiens (human),Tryptophan metabolism - Homo sapiens (human),Glycine, serine and threonine metabolism - Homo sapiens (human),Phenylalanine metabolism - Homo sapiens (human),Histidine metabolism - Homo sapiens (human),Arginine and proline metabolism - Homo sapiens (human)

45. **Predicted interaction: Sumatriptan, Tizanidine, biliary tract disorder**

- For drug Sumatriptan:
  - Proteins: 5-hydroxytryptamine receptor 1A,Amine oxidase [flavin-containing] A,5-hydroxytryptamine receptor 1D,5-hydroxytryptamine receptor 1B,5-hydroxytryptamine receptor 1F,Solute carrier organic anion transporter family member 1A2,Broad substrate specificity ATP-binding cassette transporter ABCG2,Solute carrier organic anion transporter family member 1B1
  - Pathways: Tyrosine metabolism - Homo sapiens (human),Tryptophan metabolism - Homo sapiens (human),Glycine, serine and threonine metabolism - Homo sapiens (human),Phenylalanine metabolism - Homo sapiens (human),Histidine metabolism - Homo sapiens (human),Arginine and proline metabolism - Homo sapiens (human)
- For drug Tizanidine:
  - Proteins: Alpha-2A adrenergic receptor,Alpha-2B adrenergic receptor,Alpha-2C adrenergic receptor,Nischarin
  - Pathways: Unknown

46. **Predicted interaction: Tizanidine, Rizatriptan, biliary tract disorder**

- For drug Tizanidine:
  - Proteins: Alpha-2A adrenergic receptor,Alpha-2B adrenergic receptor,Alpha-2C adrenergic receptor,Nischarin
  - Pathways: Unknown
- For drug Rizatriptan:
  - Proteins: Cytochrome P450 1A2,Amine oxidase [flavin-containing] A,5-hydroxytryptamine receptor 1D,5-hydroxytryptamine receptor 1B,5-hydroxytryptamine receptor 1F
  - Pathways: Taste transduction - Homo sapiens (human),Tyrosine metabolism - Homo sapiens (human),Alcoholism - Homo sapiens (human),Cocaine addiction - Homo sapiens (human),Amphetamine addiction - Homo sapiens (human),Tryptophan metabolism - Homo sapiens (human),Glycine, serine and threonine metabolism - Homo sapiens (human),Phenylalanine metabolism - Homo sapiens (human),Histidine metabolism - Homo sapiens (human),Arginine and proline metabolism - Homo sapiens (human)

47. **Predicted interaction: Verapamil, Mefenamic acid, black stools**

- For drug Verapamil:
  - Proteins: Voltage-dependent L-type calcium channel subunit beta-4,Voltage-dependent P/Q-type calcium channel subunit alpha-1A,Voltage-dependent T-type calcium channel subunit alpha-1G,Voltage-dependent L-type calcium channel subunit alpha-1F,Cytochrome P450 3A4,Voltage-dependent L-type calcium channel subunit beta-3,Voltage-dependent N-type calcium channel subunit alpha-1B,Voltage-dependent L-type calcium channel subunit beta-1,ATP-sensitive inward rectifier potassium channel 11,Voltage-dependent T-type calcium channel subunit alpha-1I
  - Pathways: Retinol metabolism - Homo sapiens (human),Drug metabolism - cytochrome P450 - Homo sapiens (human),Steroid hormone biosynthesis - Homo sapiens (human),Linoleic acid metabolism - Homo sapiens (human)
- For drug Mefenamic acid:
  - Proteins: Cytochrome P450 2C8,Prostaglandin G/H synthase 1,Prostaglandin G/H synthase 2
  - Pathways: Human papillomavirus infection - Homo sapiens (human),Kaposi sarcoma-associated herpesvirus infection - Homo sapiens (human),Human cytomegalovirus infection - Homo sapiens (human)

48. **Predicted interaction: Valdecoxib, Mefenamic acid, black stools**

- For drug Valdecoxib:
  - Proteins: Carbonic anhydrase 2,Carbonic anhydrase 3,Cytochrome P450 3A4
  - Pathways: Retinol metabolism - Homo sapiens (human),Drug metabolism - cytochrome P450 - Homo sapiens (human),Steroid hormone biosynthesis - Homo sapiens (human),Pancreatic secretion - Homo sapiens (human),Nitrogen metabolism - Homo sapiens (human),Linoleic acid metabolism - Homo sapiens (human),Gastric acid secretion - Homo sapiens (human),Proximal tubule bicarbonate reclamation - Homo sapiens (human),Collecting duct acid secretion - Homo sapiens (human)
- For drug Mefenamic acid:
  - Proteins: Cytochrome P450 2C8,Prostaglandin G/H synthase 1,Prostaglandin G/H synthase 2
  - Pathways: Human papillomavirus infection - Homo sapiens (human),Kaposi sarcoma-associated herpesvirus infection - Homo sapiens (human),Human cytomegalovirus infection - Homo sapiens (human)

49. **Predicted interaction: Tacrolimus, Sevelamer, bleb**

- For drug Tacrolimus:
  - Proteins: Peptidyl-prolyl cis-trans isomerase FKBP1A,Cholesterol transporter ABCA5
  - Pathways: ABC transporters - Homo sapiens (human)
- For drug Sevelamer:
  - Proteins:
  - Pathways: Unknown

50. **Predicted interaction: Zaleplon, Promethazine, blood calcium decreased**

- For drug Zaleplon:
  - Proteins: Cytochrome P450 3A4,Gamma-aminobutyric acid receptor subunit alpha-1,Cytochrome P450 3A7,Translocator protein,Aldehyde oxidase
  - Pathways: Nicotine addiction - Homo sapiens (human),Nicotinate and nicotinamide metabolism - Homo sapiens (human),Vitamin B6 metabolism - Homo sapiens (human)
- For drug Promethazine:

  - Proteins: Muscarinic acetylcholine receptor M5,D(2) dopamine receptor,Histamine H2 receptor,5-hydroxytryptamine receptor 2A,Histamine H1 receptor
  - Pathways: Dopaminergic synapse - Homo sapiens (human),Rap1 signaling pathway - Homo sapiens (human),Inflammatory mediator regulation of TRP channels - Homo sapiens (human),Alcoholism - Homo sapiens (human),Parkinson disease - Homo sapiens (human),Cocaine addiction - Homo sapiens (human)

51. **Predicted interaction: Orphenadrine, Sertraline, blood disorder**

- For drug Orphenadrine:
  - Proteins: Glutamate receptor ionotropic, NMDA 2D,Glutamate receptor ionotropic, NMDA 3B,Cytochrome P450 1A2,Cytochrome P450 3A4,Cytochrome P450 2B6,Sodium-dependent noradrenaline transporter,Histamine H1 receptor,Glutamate receptor ionotropic, NMDA 1,Glutamate receptor ionotropic, NMDA 3A,Sodium channel protein type 10 subunit alpha
  - Pathways: Nicotine addiction - Homo sapiens (human),Ras signaling pathway - Homo sapiens (human),Glutamatergic synapse - Homo sapiens (human),Circadian entrainment - Homo sapiens (human),Amyotrophic lateral sclerosis (ALS) - Homo sapiens (human),Alzheimer disease - Homo sapiens (human),Huntington disease - Homo sapiens (human),Long-term potentiation - Homo sapiens (human)
- For drug Sertraline:
  - Proteins: Membrane-associated progesterone receptor component 1,Sodium-dependent serotonin transporter
  - Pathways: Unknown

52. **Predicted interaction: Pamidronate, Ketorolac, bulging**

- For drug Pamidronate:
  - Proteins: Farnesyl pyrophosphate synthase
  - Pathways: Human T-cell leukemia virus 1 infection - Homo sapiens (human),Influenza A - Homo sapiens (human),Terpenoid backbone biosynthesis - Homo sapiens (human)
- For drug Ketorolac:
  - Proteins: Prostaglandin G/H synthase 2
  - Pathways: Chemical carcinogenesis - Homo sapiens (human),MicroRNAs in cancer - Homo sapiens (human),Serotonergic synapse - Homo sapiens (human),Arachidonic acid metabolism - Homo sapiens (human)

53. **Predicted interaction: Prednisone, Oxaprozin, bundle branch block right**

- For drug Prednisone:
  - Proteins: ATP-dependent translocase ABCB1,Cytochrome P450 3A4,Corticosteroid 11-beta-dehydrogenase isozyme 1,Solute carrier organic anion transporter family member 1A2
  - Pathways: Retinol metabolism - Homo sapiens (human),Metabolism of xenobiotics by cytochrome P450 - Homo sapiens (human),Drug metabolism - cytochrome P450 - Homo sapiens (human),Steroid hormone biosynthesis - Homo sapiens (human),Gastric cancer - Homo sapiens (human),MicroRNAs in cancer - Homo sapiens (human),ABC transporters - Homo sapiens (human),Bile secretion - Homo sapiens (human),Linoleic acid metabolism - Homo sapiens (human)
- For drug Oxaprozin:
  - Proteins: Cytochrome P450 2C9,Prostaglandin G/H synthase 1,Prostaglandin G/H synthase 2
  - Pathways: Small cell lung cancer - Homo sapiens (human),Human papillomavirus infection - Homo sapiens (human),Kaposi sarcoma-associated herpesvirus infection - Homo sapiens (human),C-type lectin receptor signaling pathway - Homo sapiens (human),TNF

signaling pathway - Homo sapiens (human),Human cytomegalovirus infection - Homo sapiens (human),VEGF signaling pathway - Homo sapiens (human),Leishmaniasis - Homo sapiens (human),IL-17 signaling pathway - Homo sapiens (human),NF-kappa B signaling pathway - Homo sapiens (human)

54. **Predicted interaction: Rofecoxib, Oxaprozin, bundle branch block right**
    - For drug Rofecoxib:
      - Proteins: Cytochrome P450 3A4,Cytochrome P450 2C8,Cytochrome P450 2C9,Elastin
      - Pathways: Retinol metabolism - Homo sapiens (human),Metabolism of xenobiotics by cytochrome P450 - Homo sapiens (human),Drug metabolism - cytochrome P450 - Homo sapiens (human),Steroid hormone biosynthesis - Homo sapiens (human),Bile secretion - Homo sapiens (human),Linoleic acid metabolism - Homo sapiens (human)
    - For drug Oxaprozin:
      - Proteins: Cytochrome P450 2C9,Prostaglandin G/H synthase 1,Prostaglandin G/H synthase 2
      - Pathways: Small cell lung cancer - Homo sapiens (human),Human papillomavirus infection - Homo sapiens (human),Kaposi sarcoma-associated herpesvirus infection - Homo sapiens (human),C-type lectin receptor signaling pathway - Homo sapiens (human),TNF signaling pathway - Homo sapiens (human),Human cytomegalovirus infection - Homo sapiens (human),VEGF signaling pathway - Homo sapiens (human),Leishmaniasis - Homo sapiens (human),IL-17 signaling pathway - Homo sapiens (human),NF-kappa B signaling pathway - Homo sapiens (human)
55. **Predicted interaction: Celecoxib, Oxaprozin, bundle branch block right**
    - For drug Celecoxib:
      - Proteins: 3-phosphoinositide-dependent protein kinase 1,Cytochrome P450 3A4,Cytochrome P450 2C8,Cytochrome P450 2D6,Cytochrome P450 2C9,ATP-binding cassette sub-family B member 5
      - Pathways: Retinol metabolism - Homo sapiens (human),Metabolism of xenobiotics by cytochrome P450 - Homo sapiens (human),Drug metabolism - cytochrome P450 - Homo sapiens (human),Steroid hormone biosynthesis - Homo sapiens (human),ABC transporters - Homo sapiens (human),Bile secretion - Homo sapiens (human),Linoleic acid metabolism - Homo sapiens (human)
    - For drug Oxaprozin:
      - Proteins: Cytochrome P450 2C9,Prostaglandin G/H synthase 1,Prostaglandin G/H synthase 2
      - Pathways: Small cell lung cancer - Homo sapiens (human),Human papillomavirus infection - Homo sapiens (human),Kaposi sarcoma-associated herpesvirus infection - Homo sapiens (human),C-type lectin receptor signaling pathway - Homo sapiens (human),TNF signaling pathway - Homo sapiens (human),Human cytomegalovirus infection - Homo sapiens (human),VEGF signaling pathway - Homo sapiens (human),Leishmaniasis - Homo sapiens (human),IL-17 signaling pathway - Homo sapiens (human),NF-kappa B signaling pathway - Homo sapiens (human)
56. **Predicted interaction: Ciprofloxacin, Oxaprozin, bundle branch block right**
    - For drug Ciprofloxacin:
      - Proteins: ATP-dependent translocase ABCB1,Cytochrome P450 3A4,DNA gyrase subunit A,DNA topoisomerase 4 subunit A
      - Pathways: Retinol metabolism - Homo sapiens (human),Metabolism of xenobiotics by cytochrome P450 - Homo sapiens (human),Drug metabolism - cytochrome P450 - Homo sapiens (human),Steroid hormone biosynthesis - Homo sapiens (human),Gastric cancer - Homo sapiens (human),MicroRNAs in cancer - Homo sapiens (human),ABC transporters - Homo sapiens (human),Bile secretion - Homo sapiens (human),Linoleic acid metabolism - Homo sapiens (human)

- For drug Oxaprozin:
  - Proteins: Cytochrome P450 2C9,Prostaglandin G/H synthase 1,Prostaglandin G/H synthase 2
  - Pathways: Small cell lung cancer - Homo sapiens (human),Human papillomavirus infection - Homo sapiens (human),Kaposi sarcoma-associated herpesvirus infection - Homo sapiens (human),C-type lectin receptor signaling pathway - Homo sapiens (human),TNF signaling pathway - Homo sapiens (human),Human cytomegalovirus infection - Homo sapiens (human),VEGF signaling pathway - Homo sapiens (human),Leishmaniasis - Homo sapiens (human),IL-17 signaling pathway - Homo sapiens (human),NF-kappa B signaling pathway - Homo sapiens (human)

57. **Predicted interaction: Oxaprozin, Venlafaxine, cardiac decompensation**
    - For drug Oxaprozin:
      - Proteins: Cytochrome P450 2C9,Prostaglandin G/H synthase 1,Prostaglandin G/H synthase 2
      - Pathways: Small cell lung cancer - Homo sapiens (human),Human papillomavirus infection - Homo sapiens (human),Kaposi sarcoma-associated herpesvirus infection - Homo sapiens (human),C-type lectin receptor signaling pathway - Homo sapiens (human),TNF signaling pathway - Homo sapiens (human),Human cytomegalovirus infection - Homo sapiens (human),VEGF signaling pathway - Homo sapiens (human),Leishmaniasis - Homo sapiens (human),IL-17 signaling pathway - Homo sapiens (human),NF-kappa B signaling pathway - Homo sapiens (human)
    - For drug Venlafaxine:
      - Proteins: Sodium-dependent dopamine transporter
      - Pathways: Dopaminergic synapse - Homo sapiens (human),Alcoholism - Homo sapiens (human),Parkinson disease - Homo sapiens (human),Cocaine addiction - Homo sapiens (human)

58. **Predicted interaction: Prednisone, Ticlopidine, cardiac ischemia**
    - For drug Prednisone:
      - Proteins: ATP-dependent translocase ABCB1,Cytochrome P450 3A4,Corticosteroid 11-beta-dehydrogenase isozyme 1,Solute carrier organic anion transporter family member 1A2
      - Pathways: Retinol metabolism - Homo sapiens (human),Metabolism of xenobiotics by cytochrome P450 - Homo sapiens (human),Drug metabolism - cytochrome P450 - Homo sapiens (human),Steroid hormone biosynthesis - Homo sapiens (human),Gastric cancer - Homo sapiens (human),MicroRNAs in cancer - Homo sapiens (human),ABC transporters - Homo sapiens (human),Bile secretion - Homo sapiens (human),Linoleic acid metabolism - Homo sapiens (human)
    - For drug Ticlopidine:
      - Proteins: Myeloperoxidase,P2Y purinoceptor 12
      - Pathways: Phagosome - Homo sapiens (human),Acute myeloid leukemia - Homo sapiens (human)

59. **Predicted interaction: Prednisone, Nefazodone, cardiac ischemia**
    - For drug Prednisone:
      - Proteins: ATP-dependent translocase ABCB1,Cytochrome P450 3A4,Corticosteroid 11-beta-dehydrogenase isozyme 1,Solute carrier organic anion transporter family member 1A2
      - Pathways: Retinol metabolism - Homo sapiens (human),Metabolism of xenobiotics by cytochrome P450 - Homo sapiens (human),Drug metabolism - cytochrome P450 - Homo sapiens (human),Steroid hormone biosynthesis - Homo sapiens (human),Gastric cancer - Homo sapiens (human),MicroRNAs in cancer - Homo sapiens (human),ABC transporters - Homo sapiens (human),Bile secretion - Homo sapiens (human),Linoleic acid metabolism - Homo sapiens (human)

- For drug Nefazodone:
  - Proteins: Sodium-dependent noradrenaline transporter,Sodium-dependent serotonin transporter,Sodium-dependent dopamine transporter,CYP2B protein
  - Pathways: Alcoholism - Homo sapiens (human),Parkinson disease - Homo sapiens (human),Cocaine addiction - Homo sapiens (human),Amphetamine addiction - Homo sapiens (human)

60. **Predicted interaction: Cimetidine, Lovastatin, cardiomyopathy**

- For drug Cimetidine:
  - Proteins: Solute carrier family 22 member 3,Cytochrome P450 11B1, mitochondrial,Cytochrome P450 3A7,Histamine H2 receptor,Dimethylaniline monooxygenase [N-oxide-forming] 3,Dimethylaniline monooxygenase [N-oxide-forming] 1,Multidrug and toxin extrusion protein 1,Solute carrier family 22 member 4
  - Pathways: Cortisol synthesis and secretion - Homo sapiens (human),Cushing syndrome - Homo sapiens (human),Gastric acid secretion - Homo sapiens (human)
- For drug Lovastatin:
  - Proteins: 3-hydroxy-3-methylglutaryl-coenzyme A reductase,Integrin alpha-L,Serum paraoxonase/lactonase 3,Histone deacetylase 2
  - Pathways: Viral myocarditis - Homo sapiens (human),Leukocyte transendothelial migration - Homo sapiens (human),Natural killer cell mediated cytotoxicity - Homo sapiens (human),Staphylococcus aureus infection - Homo sapiens (human),Cell adhesion molecules (CAMs) - Homo sapiens (human),Malaria - Homo sapiens (human),Cell cycle - Homo sapiens (human),Notch signaling pathway - Homo sapiens (human)

61. **Predicted interaction: Cimetidine, Nadolol, cardiomyopathy**

- For drug Cimetidine:
  - Proteins: Solute carrier family 22 member 3,Cytochrome P450 11B1, mitochondrial,Cytochrome P450 3A7,Histamine H2 receptor,Dimethylaniline monooxygenase [N-oxide-forming] 3,Dimethylaniline monooxygenase [N-oxide-forming] 1,Multidrug and toxin extrusion protein 1,Solute carrier family 22 member 4
  - Pathways: Cortisol synthesis and secretion - Homo sapiens (human),Cushing syndrome - Homo sapiens (human),Gastric acid secretion - Homo sapiens (human)
- For drug Nadolol:
  - Proteins:
  - Pathways: Unknown

62. **Predicted interaction: Cimetidine, Nadolol, cardiomyopathy**

- For drug Cimetidine:
  - Proteins: Solute carrier family 22 member 3,Cytochrome P450 11B1, mitochondrial,Cytochrome P450 3A7,Histamine H2 receptor,Dimethylaniline monooxygenase [N-oxide-forming] 3,Dimethylaniline monooxygenase [N-oxide-forming] 1,Multidrug and toxin extrusion protein 1,Solute carrier family 22 member 4
  - Pathways: Cortisol synthesis and secretion - Homo sapiens (human),Cushing syndrome - Homo sapiens (human),Gastric acid secretion - Homo sapiens (human)
- For drug Nadolol:
  - Proteins:
  - Pathways: Unknown

63. **Predicted interaction: Estazolam, Zolpidem, cerebral artery embolism**

- For drug Estazolam:
  - Proteins:

- Pathways: Unknown

- For drug Zolpidem:
  - Proteins: Cytochrome P450 3A4,Cytochrome P450 2D6,Cytochrome P450 2C9
  - Pathways: Retinol metabolism - Homo sapiens (human),Metabolism of xenobiotics by cytochrome P450 - Homo sapiens (human),Drug metabolism - cytochrome P450 - Homo sapiens (human),Steroid hormone biosynthesis - Homo sapiens (human),Bile secretion - Homo sapiens (human),Linoleic acid metabolism - Homo sapiens (human)

64. **Predicted interaction: Estazolam, Oxycodone, cerebral artery embolism**
    - For drug Estazolam:
      - Proteins:
      - Pathways: Unknown
    - For drug Oxycodone:
      - Proteins: Cytochrome P450 3A4,Cytochrome P450 2D6
      - Pathways: Retinol metabolism - Homo sapiens (human),Metabolism of xenobiotics by cytochrome P450 - Homo sapiens (human),Drug metabolism - cytochrome P450 - Homo sapiens (human),Steroid hormone biosynthesis - Homo sapiens (human),Bile secretion - Homo sapiens (human),Linoleic acid metabolism - Homo sapiens (human)

65. **Predicted interaction: Rofecoxib, Alosetron, cerebral ischaemia**
    - For drug Rofecoxib:
      - Proteins: Cytochrome P450 3A4,Cytochrome P450 2C8,Cytochrome P450 2C9,Elastin
      - Pathways: Retinol metabolism - Homo sapiens (human),Metabolism of xenobiotics by cytochrome P450 - Homo sapiens (human),Drug metabolism - cytochrome P450 - Homo sapiens (human),Steroid hormone biosynthesis - Homo sapiens (human),Bile secretion - Homo sapiens (human),Linoleic acid metabolism - Homo sapiens (human)
    - For drug Alosetron:
      - Proteins: 5-hydroxytryptamine receptor 3A
      - Pathways: Taste transduction - Homo sapiens (human)

66. **Predicted interaction: Ciprofloxacin, Alosetron, cerebral ischaemia**
    - For drug Ciprofloxacin:
      - Proteins: ATP-dependent translocase ABCB1,Cytochrome P450 3A4,DNA gyrase subunit A,DNA topoisomerase 4 subunit A
      - Pathways: Retinol metabolism - Homo sapiens (human),Metabolism of xenobiotics by cytochrome P450 - Homo sapiens (human),Drug metabolism - cytochrome P450 - Homo sapiens (human),Steroid hormone biosynthesis - Homo sapiens (human),Gastric cancer - Homo sapiens (human),MicroRNAs in cancer - Homo sapiens (human),ABC transporters - Homo sapiens (human),Bile secretion - Homo sapiens (human),Linoleic acid metabolism - Homo sapiens (human)
    - For drug Alosetron:
      - Proteins: 5-hydroxytryptamine receptor 3A
      - Pathways: Taste transduction - Homo sapiens (human)

67. **Predicted interaction: Ketorolac, Oxaprozin, cervical dysplasia**
    - For drug Ketorolac:
      - Proteins:
      - Pathways: Unknown
    - For drug Oxaprozin:
      - Proteins: Cytochrome P450 2C9,Prostaglandin G/H synthase 1,Prostaglandin G/H synthase 2

- Pathways: Small cell lung cancer - Homo sapiens (human),Human papillomavirus infection - Homo sapiens (human),Kaposi sarcoma-associated herpesvirus infection - Homo sapiens (human),C-type lectin receptor signaling pathway - Homo sapiens (human),TNF signaling pathway - Homo sapiens (human),Human cytomegalovirus infection - Homo sapiens (human),VEGF signaling pathway - Homo sapiens (human),Leishmaniasis - Homo sapiens (human),IL-17 signaling pathway - Homo sapiens (human),NF-kappa B signaling pathway - Homo sapiens (human)

68. **Predicted interaction: Rofecoxib, Pamidronate, coronary angioplasty**

- For drug Rofecoxib:
  - Proteins: Cytochrome P450 2C9,Elastin,Prostaglandin G/H synthase 2
  - Pathways: Chemical carcinogenesis - Homo sapiens (human),Drug metabolism - cytochrome P450 - Homo sapiens (human),MicroRNAs in cancer - Homo sapiens (human),Serotonergic synapse - Homo sapiens (human),Arachidonic acid metabolism - Homo sapiens (human),Linoleic acid metabolism - Homo sapiens (human)
- For drug Pamidronate:
  - Proteins: Farnesyl pyrophosphate synthase
  - Pathways: Human T-cell leukemia virus 1 infection - Homo sapiens (human),Influenza A - Homo sapiens (human),Terpenoid backbone biosynthesis - Homo sapiens (human)

69. **Predicted interaction: Thiothixene, Zolpidem, cystitis interstitial**

- For drug Thiothixene:
  - Proteins: D(2) dopamine receptor,D(1A) dopamine receptor,5-hydroxytryptamine receptor 2A
  - Pathways: Gap junction - Homo sapiens (human),Dopaminergic synapse - Homo sapiens (human),Rap1 signaling pathway - Homo sapiens (human),Calcium signaling pathway - Homo sapiens (human),Inflammatory mediator regulation of TRP channels - Homo sapiens (human),Alcoholism - Homo sapiens (human),Parkinson disease - Homo sapiens (human),Cocaine addiction - Homo sapiens (human),Amphetamine addiction - Homo sapiens (human)
- For drug Zolpidem:
  - Proteins: Cytochrome P450 3A4,Cytochrome P450 2D6,Cytochrome P450 2C9
  - Pathways: Retinol metabolism - Homo sapiens (human),Metabolism of xenobiotics by cytochrome P450 - Homo sapiens (human),Drug metabolism - cytochrome P450 - Homo sapiens (human),Steroid hormone biosynthesis - Homo sapiens (human),Bile secretion - Homo sapiens (human),Linoleic acid metabolism - Homo sapiens (human)

70. **Predicted interaction: Thiothixene, Oxycodone, cystitis interstitial**

- For drug Thiothixene:
  - Proteins: D(2) dopamine receptor,D(1A) dopamine receptor,5-hydroxytryptamine receptor 2A
  - Pathways: Gap junction - Homo sapiens (human),Dopaminergic synapse - Homo sapiens (human),Rap1 signaling pathway - Homo sapiens (human),Calcium signaling pathway - Homo sapiens (human),Inflammatory mediator regulation of TRP channels - Homo sapiens (human),Alcoholism - Homo sapiens (human),Parkinson disease - Homo sapiens (human),Cocaine addiction - Homo sapiens (human),Amphetamine addiction - Homo sapiens (human)
- For drug Oxycodone:
  - Proteins: Cytochrome P450 3A4,Cytochrome P450 2D6
  - Pathways: Retinol metabolism - Homo sapiens (human),Metabolism of xenobiotics by cytochrome P450 - Homo sapiens (human),Drug metabolism - cytochrome P450 - Homo sapiens (human),Steroid hormone biosynthesis - Homo sapiens (human),Bile secretion - Homo sapiens (human),Linoleic acid metabolism - Homo sapiens (human)

71. **Predicted interaction: Venlafaxine, Rizatriptan, duodenitis**

- For drug Venlafaxine:
  - Proteins: Sodium-dependent dopamine transporter
  - Pathways: Dopaminergic synapse - Homo sapiens (human),Alcoholism - Homo sapiens (human),Parkinson disease - Homo sapiens (human),Cocaine addiction - Homo sapiens (human)
- For drug Rizatriptan:
  - Proteins: Cytochrome P450 1A2,Amine oxidase [flavin-containing] A,5-hydroxytryptamine receptor 1D,5-hydroxytryptamine receptor 1B,5-hydroxytryptamine receptor 1F
  - Pathways: Taste transduction - Homo sapiens (human),Tyrosine metabolism - Homo sapiens (human),Alcoholism - Homo sapiens (human),Cocaine addiction - Homo sapiens (human),Amphetamine addiction - Homo sapiens (human),Tryptophan metabolism - Homo sapiens (human),Glycine, serine and threonine metabolism - Homo sapiens (human),Phenylalanine metabolism - Homo sapiens (human),Histidine metabolism - Homo sapiens (human),Arginine and proline metabolism - Homo sapiens (human)

72. **Predicted interaction: Oxycodone, Nefazodone, dyspnoea paroxysmal nocturnal**

- For drug Oxycodone:
  - Proteins: Cytochrome P450 3A4,Cytochrome P450 2D6
  - Pathways: Retinol metabolism - Homo sapiens (human),Metabolism of xenobiotics by cytochrome P450 - Homo sapiens (human),Drug metabolism - cytochrome P450 - Homo sapiens (human),Steroid hormone biosynthesis - Homo sapiens (human),Bile secretion - Homo sapiens (human),Linoleic acid metabolism - Homo sapiens (human)
- For drug Nefazodone:
  - Proteins: CYP2B protein
  - Pathways: Unknown

73. **Predicted interaction: Nefazodone, Temazepam, dyspnoea paroxysmal nocturnal**

- For drug Nefazodone:
  - Proteins: CYP2B protein
  - Pathways: Unknown
- For drug Temazepam:
  - Proteins: Cytochrome P450 3A4,Translocator protein
  - Pathways: Neuroactive ligand-receptor interaction - Homo sapiens (human),Chemical carcinogenesis - Homo sapiens (human),Retinol metabolism - Homo sapiens (human),Metabolism of xenobiotics by cytochrome P450 - Homo sapiens (human),Drug metabolism - cytochrome P450 - Homo sapiens (human),Bile secretion - Homo sapiens (human),Linoleic acid metabolism - Homo sapiens (human)

74. **Predicted interaction: Piroxicam, Sulfasalazine, dysuria**

- For drug Piroxicam:
  - Proteins: Prostaglandin G/H synthase 1,Prostaglandin G/H synthase 2
  - Pathways: Small cell lung cancer - Homo sapiens (human),VEGF signaling pathway - Homo sapiens (human),Leishmaniasis - Homo sapiens (human),IL-17 signaling pathway - Homo sapiens (human),NF-kappa B signaling pathway - Homo sapiens (human)
- For drug Sulfasalazine:
  - Proteins: Inhibitor of nuclear factor kappa-B kinase subunit beta,Inhibitor of nuclear factor kappa-B kinase subunit alpha,Phospholipase A2,Polyunsaturated fatty acid 5-lipoxygenase,Prostaglandin G/H synthase 1,Thromboxane-A synthase,Acetyl-CoA acetyltransferase, mitochondrial,Prostaglandin G/H synthase 2,Peroxisome proliferator-activated receptor gamma,Proton-coupled folate transporter,Cystine/glutamate transporter

- Pathways: Small cell lung cancer - Homo sapiens (human),VEGF signaling pathway - Homo sapiens (human),Glyoxylate and dicarboxylate metabolism - Homo sapiens (human),Butanoate metabolism - Homo sapiens (human),Propanoate metabolism - Homo sapiens (human),Leishmaniasis - Homo sapiens (human),IL-17 signaling pathway - Homo sapiens (human),NF-kappa B signaling pathway - Homo sapiens (human),Fatty acid metabolism - Homo sapiens (human),Synthesis and degradation of ketone bodies - Homo sapiens (human)

75. **Predicted interaction: Ropinirole, Zaleplon, edema extremities**
    - For drug Ropinirole:
      - Proteins: Cytochrome P450 1A2,Cytochrome P450 3A4
      - Pathways: Chemical carcinogenesis - Homo sapiens (human),Retinol metabolism - Homo sapiens (human),Metabolism of xenobiotics by cytochrome P450 - Homo sapiens (human),Drug metabolism - cytochrome P450 - Homo sapiens (human),Bile secretion - Homo sapiens (human),Linoleic acid metabolism - Homo sapiens (human)
    - For drug Zaleplon:
      - Proteins:
      - Pathways: Unknown
76. **Predicted interaction: Nadolol, Oxycodone, emesis**
    - For drug Nadolol:
      - Proteins: Beta-2 adrenergic receptor,Beta-1 adrenergic receptor
      - Pathways: Unknown
    - For drug Oxycodone:
      - Proteins: Alpha-1-acid glycoprotein 1,Mu-type opioid receptor,Delta-type opioid receptor,Kappa-type opioid receptor
      - Pathways: Neuroactive ligand-receptor interaction - Homo sapiens (human),Sphingolipid signaling pathway - Homo sapiens (human),Estrogen signaling pathway - Homo sapiens (human),Morphine addiction - Homo sapiens (human)
77. **Predicted interaction: Fentanyl, Naratriptan, endocarditis**
    - For drug Fentanyl:
      - Proteins:
      - Pathways: Unknown
    - For drug Naratriptan:
      - Proteins: 5-hydroxytryptamine receptor 1A,Amine oxidase [flavin-containing] A,5-hydroxytryptamine receptor 1D,5-hydroxytryptamine receptor 1B,5-hydroxytryptamine receptor 1F
      - Pathways: Dopaminergic synapse - Homo sapiens (human),Tyrosine metabolism - Homo sapiens (human),Alcoholism - Homo sapiens (human),Cocaine addiction - Homo sapiens (human),Amphetamine addiction - Homo sapiens (human),Tryptophan metabolism - Homo sapiens (human),Glycine, serine and threonine metabolism - Homo sapiens (human),Phenylalanine metabolism - Homo sapiens (human),Histidine metabolism - Homo sapiens (human),Arginine and proline metabolism - Homo sapiens (human)
78. **Predicted interaction: Mirtazapine, Naratriptan, endocarditis**
    - For drug Mirtazapine:
      - Proteins: D(2) dopamine receptor,5-hydroxytryptamine receptor 2A,Sodium-dependent serotonin transporter,Histamine H1 receptor,Histamine H3 receptor
      - Pathways: Dopaminergic synapse - Homo sapiens (human),Rap1 signaling pathway - Homo sapiens (human),Inflammatory mediator regulation of TRP channels - Homo sapiens (human),Alcoholism - Homo sapiens (human),Parkinson disease - Homo sapiens (human),Cocaine addiction - Homo sapiens (human)

- For drug Naratriptan:
  - Proteins: 5-hydroxytryptamine receptor 1A,Amine oxidase [flavin-containing] A,5-hydroxytryptamine receptor 1D,5-hydroxytryptamine receptor 1B,5-hydroxytryptamine receptor 1F
  - Pathways: Dopaminergic synapse - Homo sapiens (human),Tyrosine metabolism - Homo sapiens (human),Alcoholism - Homo sapiens (human),Cocaine addiction - Homo sapiens (human),Amphetamine addiction - Homo sapiens (human),Tryptophan metabolism - Homo sapiens (human),Glycine, serine and threonine metabolism - Homo sapiens (human),Phenylalanine metabolism - Homo sapiens (human),Histidine metabolism - Homo sapiens (human),Arginine and proline metabolism - Homo sapiens (human)

79. **Predicted interaction: Clonazepam, Methocarbamol, enlarged liver**
    - For drug Clonazepam:
      - Proteins: Nuclear receptor subfamily 1 group I member 2,Cytochrome P450 3A4,Arylamine N-acetyltransferase 2,Gamma-aminobutyric acid receptor subunit alpha-1,Translocator protein
      - Pathways: Neuroactive ligand-receptor interaction - Homo sapiens (human),Metabolism of xenobiotics by cytochrome P450 - Homo sapiens (human),Drug metabolism - cytochrome P450 - Homo sapiens (human),Bile secretion - Homo sapiens (human)
    - For drug Methocarbamol:
      - Proteins: Carbonic anhydrase 1
      - Pathways: Nitrogen metabolism - Homo sapiens (human)

80. **Predicted interaction: Paroxetine, Methocarbamol, enlarged liver**
    - For drug Paroxetine:
      - Proteins: ATP-dependent translocase ABCB1,Muscarinic acetylcholine receptor M5,Cytochrome P450 2C8,Cytochrome P450 2D6
      - Pathways: Neuroactive ligand-receptor interaction - Homo sapiens (human),Metabolism of xenobiotics by cytochrome P450 - Homo sapiens (human),Drug metabolism - cytochrome P450 - Homo sapiens (human),Gastric cancer - Homo sapiens (human),MicroRNAs in cancer - Homo sapiens (human),Bile secretion - Homo sapiens (human),Calcium signaling pathway - Homo sapiens (human),Arachidonic acid metabolism - Homo sapiens (human)
    - For drug Methocarbamol:
      - Proteins: Carbonic anhydrase 1
      - Pathways: Nitrogen metabolism - Homo sapiens (human)

81. **Predicted interaction: Thiothixene, Metoclopramide, enterocolitis**
    - For drug Thiothixene:
      - Proteins: D(2) dopamine receptor,D(1A) dopamine receptor,5-hydroxytryptamine receptor 2A
      - Pathways: Gap junction - Homo sapiens (human),Dopaminergic synapse - Homo sapiens (human),Rap1 signaling pathway - Homo sapiens (human),Calcium signaling pathway - Homo sapiens (human),Inflammatory mediator regulation of TRP channels - Homo sapiens (human),Alcoholism - Homo sapiens (human),Parkinson disease - Homo sapiens (human),Cocaine addiction - Homo sapiens (human),Amphetamine addiction - Homo sapiens (human)
    - For drug Metoclopramide:
      - Proteins: Cytochrome P450 2D6,5-hydroxytryptamine receptor 4
      - Pathways: Metabolism of xenobiotics by cytochrome P450 - Homo sapiens (human),Drug metabolism - cytochrome P450 - Homo sapiens (human)

82. **Predicted interaction: Oxaprozin, Piroxicam, eosinophil count increased**

- For drug Oxaprozin:
  - Proteins: Prostaglandin G/H synthase 1,Prostaglandin G/H synthase 2
  - Pathways: Small cell lung cancer - Homo sapiens (human),VEGF signaling pathway - Homo sapiens (human),Leishmaniasis - Homo sapiens (human),IL-17 signaling pathway - Homo sapiens (human),NF-kappa B signaling pathway - Homo sapiens (human)
- For drug Piroxicam:
  - Proteins: Prostaglandin G/H synthase 1,Prostaglandin G/H synthase 2
  - Pathways: Small cell lung cancer - Homo sapiens (human),VEGF signaling pathway - Homo sapiens (human),Leishmaniasis - Homo sapiens (human),IL-17 signaling pathway - Homo sapiens (human),NF-kappa B signaling pathway - Homo sapiens (human)

83. **Predicted interaction: Venlafaxine, Naratriptan, feeling unwell**

- For drug Venlafaxine:
  - Proteins: Sodium-dependent serotonin transporter
  - Pathways: Unknown
- For drug Naratriptan:
  - Proteins: 5-hydroxytryptamine receptor 1A,Amine oxidase [flavin-containing] A,5-hydroxytryptamine receptor 1D,5-hydroxytryptamine receptor 1B,5-hydroxytryptamine receptor 1F
  - Pathways: Dopaminergic synapse - Homo sapiens (human),Tyrosine metabolism - Homo sapiens (human),Alcoholism - Homo sapiens (human),Cocaine addiction - Homo sapiens (human),Amphetamine addiction - Homo sapiens (human),Tryptophan metabolism - Homo sapiens (human),Glycine, serine and threonine metabolism - Homo sapiens (human),Phenylalanine metabolism - Homo sapiens (human),Histidine metabolism - Homo sapiens (human),Arginine and proline metabolism - Homo sapiens (human)

84. **Predicted interaction: Pamidronate, Nabumetone, gingivitis**

- For drug Pamidronate:
  - Proteins: Farnesyl pyrophosphate synthase
  - Pathways: Human T-cell leukemia virus 1 infection - Homo sapiens (human),Influenza A - Homo sapiens (human),Terpenoid backbone biosynthesis - Homo sapiens (human)
- For drug Nabumetone:
  - Proteins: Prostaglandin G/H synthase 2
  - Pathways: Chemical carcinogenesis - Homo sapiens (human),MicroRNAs in cancer - Homo sapiens (human),Serotonergic synapse - Homo sapiens (human),Arachidonic acid metabolism - Homo sapiens (human)

85. **Predicted interaction: Pramipexole, Buspirone, glucose intolerance**

- For drug Pramipexole:
  - Proteins:
  - Pathways: Unknown
- For drug Buspirone:
  - Proteins: 5-hydroxytryptamine receptor 1A,Cytochrome P450 2D6,D(2) dopamine receptor,Cytochrome P450 3A5,Cytochrome P450 3A7
  - Pathways: Chemical carcinogenesis - Homo sapiens (human),Drug metabolism - cytochrome P450 - Homo sapiens (human),Endocrine resistance - Homo sapiens (human),Taste transduction - Homo sapiens (human),Serotonergic synapse - Homo sapiens (human)

86. **Predicted interaction: Zaleplon, Pregabalin, haemangioma**

- For drug Zaleplon:
  - Proteins: Cytochrome P450 3A4,Gamma-aminobutyric acid receptor subunit alpha-1,Cytochrome P450 3A7,Translocator protein,Aldehyde oxidase

  - Pathways: Nicotine addiction - Homo sapiens (human),Nicotinate and nicotinamide metabolism - Homo sapiens (human),Vitamin B6 metabolism - Homo sapiens (human)
- For drug Pregabalin:
  - Proteins: Voltage-dependent P/Q-type calcium channel subunit alpha-1A,Excitatory amino acid transporter 3
  - Pathways: Dopaminergic synapse - Homo sapiens (human)

87. **Predicted interaction: Famotidine, Oxaprozin, labyrinthitis**
   - For drug Famotidine:
     - Proteins:
     - Pathways: Unknown
   - For drug Oxaprozin:
     - Proteins: Cytochrome P450 2C9,Prostaglandin G/H synthase 1,Prostaglandin G/H synthase 2
     - Pathways: Small cell lung cancer - Homo sapiens (human),Human papillomavirus infection - Homo sapiens (human),Kaposi sarcoma-associated herpesvirus infection - Homo sapiens (human),C-type lectin receptor signaling pathway - Homo sapiens (human),TNF signaling pathway - Homo sapiens (human),Human cytomegalovirus infection - Homo sapiens (human),VEGF signaling pathway - Homo sapiens (human),Leishmaniasis - Homo sapiens (human),IL-17 signaling pathway - Homo sapiens (human),NF-kappa B signaling pathway - Homo sapiens (human)

88. **Predicted interaction: Thiothixene, Venlafaxine, leucocytosis**
   - For drug Thiothixene:
     - Proteins: D(2) dopamine receptor,D(1A) dopamine receptor,5-hydroxytryptamine receptor 2A
     - Pathways: Gap junction - Homo sapiens (human),Dopaminergic synapse - Homo sapiens (human),Rap1 signaling pathway - Homo sapiens (human),Calcium signaling pathway - Homo sapiens (human),Inflammatory mediator regulation of TRP channels - Homo sapiens (human),Alcoholism - Homo sapiens (human),Parkinson disease - Homo sapiens (human),Cocaine addiction - Homo sapiens (human),Amphetamine addiction - Homo sapiens (human)
   - For drug Venlafaxine:
     - Proteins: ATP-dependent translocase ABCB1,Cytochrome P450 3A4,Cytochrome P450 2D6,Cytochrome P450 2C9
     - Pathways: Retinol metabolism - Homo sapiens (human),Metabolism of xenobiotics by cytochrome P450 - Homo sapiens (human),Drug metabolism - cytochrome P450 - Homo sapiens (human),Steroid hormone biosynthesis - Homo sapiens (human),Gastric cancer - Homo sapiens (human),MicroRNAs in cancer - Homo sapiens (human),ABC transporters - Homo sapiens (human),Bile secretion - Homo sapiens (human),Linoleic acid metabolism - Homo sapiens (human)

89. **Predicted interaction: Mirtazapine, Thiothixene, multiple sclerosis**
   - For drug Mirtazapine:
     - Proteins: Alpha-2A adrenergic receptor,D(2) dopamine receptor,Alpha-2C adrenergic receptor,D(1A) dopamine receptor,5-hydroxytryptamine receptor 2A,5-hydroxytryptamine receptor 2C,5-hydroxytryptamine receptor 7,Alpha-1A adrenergic receptor,Histamine H1 receptor,D(3) dopamine receptor,5-hydroxytryptamine receptor 2B,5-hydroxytryptamine receptor 3A,Sodium-dependent dopamine transporter,Histamine H3 receptor
     - Pathways: Gap junction - Homo sapiens (human),AMPK signaling pathway - Homo sapiens (human),Adrenergic signaling in cardiomyocytes - Homo sapiens (human),Dopaminergic synapse - Homo sapiens (human),Rap1 signaling pathway - Homo sapiens (human),Salivary secretion - Homo sapiens (human),Inflammatory mediator regulation of TRP channels - Homo sapiens (human),Alcoholism - Homo sapiens (human),Parkinson disease - Homo sapiens (human),Cocaine addiction - Homo sapiens (human)

- For drug Thiothixene:
  - Proteins: D(2) dopamine receptor,D(1A) dopamine receptor,5-hydroxytryptamine receptor 2A
  - Pathways: Gap junction - Homo sapiens (human),Dopaminergic synapse - Homo sapiens (human),Rap1 signaling pathway - Homo sapiens (human),Calcium signaling pathway - Homo sapiens (human),Inflammatory mediator regulation of TRP channels - Homo sapiens (human),Alcoholism - Homo sapiens (human),Parkinson disease - Homo sapiens (human),Cocaine addiction - Homo sapiens (human),Amphetamine addiction - Homo sapiens (human)

90. **Predicted interaction: Nefazodone, Buspirone, nephrolithiasis**
    - For drug Nefazodone:
      - Proteins: CYP2B protein
      - Pathways: Unknown
    - For drug Buspirone:
      - Proteins: 5-hydroxytryptamine receptor 1A,Cytochrome P450 2D6,D(2) dopamine receptor,Cytochrome P450 3A5,Cytochrome P450 3A7
      - Pathways: Chemical carcinogenesis - Homo sapiens (human),Drug metabolism - cytochrome P450 - Homo sapiens (human),Endocrine resistance - Homo sapiens (human),Taste transduction - Homo sapiens (human),Serotonergic synapse - Homo sapiens (human)

91. **Predicted interaction: Nefazodone, Loratadine, nephrolithiasis**
    - For drug Nefazodone:
      - Proteins: CYP2B protein
      - Pathways: Unknown
    - For drug Loratadine:
      - Proteins: Cytochrome P450 2D6,Cytochrome P450 2C19
      - Pathways: Chemical carcinogenesis - Homo sapiens (human),Drug metabolism - cytochrome P450 - Homo sapiens (human),Endocrine resistance - Homo sapiens (human),Serotonergic synapse - Homo sapiens (human),Arachidonic acid metabolism - Homo sapiens (human),Linoleic acid metabolism - Homo sapiens (human)

92. **Predicted interaction: Erythromycin, Lincomycin, optic atrophy**
    - For drug Erythromycin:
      - Proteins: Motilin receptor,Albumin,Potassium voltage-gated channel subfamily H member 2
      - Pathways: Neuroactive ligand-receptor interaction - Homo sapiens (human)
    - For drug Lincomycin:
      - Proteins: 50S ribosomal protein L10
      - Pathways: Unknown

93. **Predicted interaction: Zaleplon, Quazepam, sleep walking**
    - For drug Zaleplon:
      - Proteins: Cytochrome P450 3A4,Translocator protein
      - Pathways: Neuroactive ligand-receptor interaction - Homo sapiens (human),Chemical carcinogenesis - Homo sapiens (human),Retinol metabolism - Homo sapiens (human),Metabolism of xenobiotics by cytochrome P450 - Homo sapiens (human),Drug metabolism - cytochrome P450 - Homo sapiens (human),Bile secretion - Homo sapiens (human),Linoleic acid metabolism - Homo sapiens (human)
    - For drug Quazepam:
      - Proteins:

- Pathways: Unknown

94. **Predicted interaction: Zaleplon, Cyclobenzaprine, stress incontinence**
    - For drug Zaleplon:
        - Proteins: Cytochrome P450 3A4,Gamma-aminobutyric acid receptor subunit alpha-1,Cytochrome P450 3A7,Translocator protein,Aldehyde oxidase
        - Pathways: Nicotine addiction - Homo sapiens (human),Nicotinate and nicotinamide metabolism - Homo sapiens (human),Vitamin B6 metabolism - Homo sapiens (human)
    - For drug Cyclobenzaprine:
        - Proteins: 5-hydroxytryptamine receptor 2A
        - Pathways: Inflammatory mediator regulation of TRP channels - Homo sapiens (human)

95. **Predicted interaction: Quazepam, Clonidine, stridor**
    - For drug Quazepam:
        - Proteins:
        - Pathways: Unknown
    - For drug Clonidine:
        - Proteins: Cytochrome P450 3A4
        - Pathways: Chemical carcinogenesis - Homo sapiens (human),Retinol metabolism - Homo sapiens (human),Metabolism of xenobiotics by cytochrome P450 - Homo sapiens (human),Drug metabolism - cytochrome P450 - Homo sapiens (human),Bile secretion - Homo sapiens (human),Linoleic acid metabolism - Homo sapiens (human)

96. **Predicted interaction: Entacapone, Carisoprodol, tenosynovitis**
    - For drug Entacapone:
        - Proteins:
        - Pathways: Unknown
    - For drug Carisoprodol:
        - Proteins: Cytochrome P450 2C19
        - Pathways: Chemical carcinogenesis - Homo sapiens (human),Drug metabolism - cytochrome P450 - Homo sapiens (human),Serotonergic synapse - Homo sapiens (human),Arachidonic acid metabolism - Homo sapiens (human),Linoleic acid metabolism - Homo sapiens (human)

97. **Predicted interaction: Tramadol, Carisoprodol, ventricular fibrillation**
    - For drug Tramadol:
        - Proteins: Mu-type opioid receptor,Delta-type opioid receptor,Kappa-type opioid receptor,Alpha-7 nicotinic cholinergic receptor subunit
        - Pathways: Neuroactive ligand-receptor interaction - Homo sapiens (human),Sphingolipid signaling pathway - Homo sapiens (human),Estrogen signaling pathway - Homo sapiens (human)
    - For drug Carisoprodol:
        - Proteins:
        - Pathways: Unknown

98. **Predicted interaction: Celecoxib, Ketoprofen, wheeze**
    - For drug Celecoxib:
        - Proteins: 3-phosphoinositide-dependent protein kinase 1,Carbonic anhydrase 2,Carbonic anhydrase 3,ATP-binding cassette sub-family B member 5

- Pathways: Sphingolipid signaling pathway - Homo sapiens (human),Nitrogen metabolism - Homo sapiens (human),Proximal tubule bicarbonate reclamation - Homo sapiens (human),Collecting duct acid secretion - Homo sapiens (human)

- For drug Ketoprofen:
  - Proteins: ATP-binding cassette sub-family C member 4,C-X-C chemokine receptor type 1,Solute carrier family 22 member 11
  - Pathways: Cytokine-cytokine receptor interaction - Homo sapiens (human),Endocytosis - Homo sapiens (human)
